# Trusting self-evaluation: predicting music and language performance from subjective ratings

**DOI:** 10.3389/fpsyg.2026.1899488

**Published:** 2026-09-21

**Authors:** Christine Groß, Markus Christiner

**Affiliations:** 1 Jazeps Vitols Latvian Academy of Music, Riga, Latvia; 2 School of Health, Education, and Social Sciences, SRH University Heidelberg, Heidelberg, Germany; 3 Department of Child and Adolescent Psychiatry, Saarland University, Homburg, Germany; 4 Music Psychology and Brain Research Section, Department of Psychology, University of Graz, Graz, Austria

**Keywords:** cross-domain associations, language ability, metacognition, musicality, music-language relationship, objective performance, self-assessment, self-evaluation

## Abstract

**Introduction:**

Subjective self-evaluation is frequently used to assess perceived musical and linguistic competence, but the extent to which such ratings correspond to objectively measured performance, particularly across domains, remains unclear.

**Methods:**

A total of 104 participants completed objective music tasks assessing singing, melodic discrimination, mistuning perception, and beat perception; language tasks assessing expressive reading, speech imitation, and auditory phonological pattern recognition; and multi-item self-evaluation scales for both domains. Associations were examined using correlation analyses, hierarchical regression models, and Bland-Altman analyses.

**Results:**

Subjective self-evaluation and objective performance were moderately associated within both domains but were not interchangeable. Objective music and language performance predicted one another more strongly than cross-domain self-evaluations. Within-domain self-evaluation contributed additional variance to the corresponding performance models. Bland-Altman analyses indicated negligible mean bias at the group level but substantial individual disagreement.

**Discussion:**

Subjective self-evaluation and objective performance capture complementary but theoretically distinct aspects of musical and linguistic ability. Objective assessment therefore remains necessary when performance itself is the target, whereas self-evaluation provides additional information about perceived competence. Comparable cross-domain association patterns are consistent with partly shared mechanisms across music and language.

## 1 Introduction

The relationship between language and music has been the subject of intensive investigation, with numerous studies demonstrating positive associations between linguistic and musical abilities. These associations are often interpreted within theoretical frameworks such as Patel's OPERA hypothesis, which specifies the conditions under which such links are most likely to emerge (Patel, 2011, 2014). Building on this framework, neuroscientific studies provide compelling evidence for shared processing mechanisms, as the acoustic signals of speech and music exhibit similarities in both temporal and spectral complexity (Ding et al., 2017; Schön et al., 2004). In line with this, research has demonstrated that musical training can enhance the neural processing of speech (Parbery-Clark et al., 2012; Intartaglia et al., 2017; Besson et al., 2011). Furthermore, musical abilities have been linked to duration perception in speech (Chobert et al., 2014) and speech segmentation skills (François et al., 2013), as well as enhanced subcortical and cortical processing of speech sounds in musicians (Tierney and Kraus, 2014; Musacchia et al., 2008). Beyond neural processing, behavioral research has further illuminated the multifaceted overlaps between language and music, examining a wide range of aspects that characterize their relationship (Christiner and Reiterer, 2019).

For instance, singing ability has been closely linked to various oral language skills, including reading in one's native language (Gordon et al., 2015; Seither-Preisler et al., 2014; Christiner et al., 2026) and in a foreign language (Partanen et al., 2022), pronunciation of foreign languages, imitation of specific accents (Christiner and Reiterer, 2013, 2015), as well as vocabulary retention and recall (Ludke et al., 2014). These associations are likely attributable to the sensorimotor demands of singing (Christiner et al., 2022b; Zatorre et al., 2007). The melodic component of singing has been suggested to facilitate memory (Wallace, 1994), but melody also plays a significant role in attentional processes in both music (Besson et al., 2011) and language (Christiner et al., 2021, 2026). Rhythmic abilities, in contrast, appear to relate more strongly to phonological processing and reading comprehension (Gordon et al., 2015; Tierney and Kraus, 2014; Tierney et al., 2013), suggesting that distinct musical abilities may differentially relate to specific language domains.

On the other hand, individuals with strong musical skills, such as instrumentalists, possess superior auditory abilities (Besson et al., 2011; François et al., 2013; Kraus and Chandrasekaran, 2010), which benefit various aspects of speech processing. These include improved pitch discrimination (Christiner et al., 2022b), more effective speech segmentation (François et al., 2013), better perception of non-native tones (Alexander et al., 2005), and enhanced recognition of auditory phonological patterns (Christiner and Groß, 2025). Notably, previous research indicated that perceptual musicality measures were more strongly associated with perceptual language measures, whereas singing played a comparatively minor role (Christiner, 2020; Christiner et al., 2022a).

A further, and particularly relevant, point is that the majority of studies focus on the influence of musical abilities and/or musical training on language, whereas the potential impact of linguistic skills on musical proficiency has received considerably less empirical attention. This asymmetry warrants closer examination. First, speaking—whether in one's native or a foreign language—occurs far more frequently than engaging in musical training. Given that musical training is nevertheless one of the most prominent variables associated with enhanced language abilities, this imbalance in research focus appears questionable. Second, there is a tendency to overemphasize the effects of musical training on language while neglecting potential influences arising from innate predispositions (Schellenberg and Lima, 2024). This perspective also aligns with evidence suggesting that the relationship between language and music may be driven by shared domain-general mechanisms or stable individual differences affecting performance in both domains (Swaminathan and Schellenberg, 2020; Kragness et al., 2021). These considerations suggest that the music–language relationship should be examined bidirectionally, because it may reflect shared domain-general mechanisms that would predict associations in both directions, as well as possible reciprocal influences between language and music, rather than modeling the relationship as a unidirectional transfer from music to language.

Building on the extensive body of research highlighting the multifaceted connections between music and language, there are, among many considerations, four crucial aspects for this study. First, research has shown that perceptual and somatosensory abilities in music are associated with language-related skills. Therefore, when musical performance is assessed at the domain level, composite scores integrating key musical abilities—such as melodic and rhythmic perception as well as singing—can provide a broader representation of musical performance. This broader representation does not imply that the individual musical abilities contribute equally to each language-related skill. Rather, the composite score was intended to capture domain-level musical performance for the present cross-domain analyses. This approach increases construct coverage but necessarily reduces information about potentially differential associations between specific musical and language abilities.

Second, similar considerations apply to the measurement of language ability. However, measuring individual differences in language ability is complex because language encompasses multiple interrelated but also partly distinct abilities. In this study we combined three complementary measures, namely auditory phonological pattern recognition, imitation of unfamiliar speech, and expressive reading in the native language into a language composite score. This decision was informed by three factors: (a) the nature of the language measures and their conceptual similarity to the musicality measures, (b) their associations with musicality reported in previous research, and (c) our aim to derive a composite score that captures individual differences in language aptitude and achievement while incorporating both auditory processing and speech production.

To begin with, these language measures conceptually overlap with musical ability tasks. Auditory phonological pattern recognition parallels musicality measures in that both require participants to process novel auditory stimuli and compare temporally organized auditory units. Musicality measures often use short melodic patterns, whereas auditory phonological pattern recognition tasks use multisyllabic speech units. Both types of tasks require sound classification, pattern recognition, and auditory memory (Christiner, 2020; Christiner and Groß, 2025). Speech imitation and singing both require the generation and reproduction of sound patterns and may therefore rely on partly overlapping auditory–motor and vocal–motor processes (Christiner and Reiterer, 2013, 2015; Christiner et al., 2022a,c; Christiner and Ludke, 2026). Expressive reading shares important features with musical performance, as readers use pitch, rhythm, intensity, and phrasing to engage an audience and convey meaning (Christiner et al., 2026).

Consistent with these conceptual overlaps, all three measures have been associated with musicality in previous studies. Whereas auditory phonological pattern recognition was associated with several perceptual musicality measures (Christiner, 2020; Groß et al., 2023; Christiner and Groß, 2025), speech imitation has been linked to both perceptual musical ability and, more specifically, singing ability (Christiner and Reiterer, 2013, 2015; Coumel et al., 2023; Christiner et al., 2022a,b,c). Expressive reading, in turn, has been associated particularly strongly with melodic musical abilities, including singing performance (Groß and Christiner, 2026; Christiner et al., 2026).

From the perspective of language assessment, we aimed to include measures of both language aptitude (Meara, 2005) and language achievement. The first two used unfamiliar speech material: one assessed the perception and analysis of novel phonological patterns, whereas the other additionally required their vocal reproduction. Together, they captured related but partly dissociable perceptual and productive abilities (Golestani and Pallier, 2007; Nasir and Ostry, 2009; Christiner and Reiterer, 2015). Expressive reading complemented these tasks by assessing the behavioral expression of language-related abilities in an established linguistic system and thus participants' already developed language skills. The resulting language summary index was intended to capture individual differences across complementary language ability.

Third, since the overlap between music and language is likely not strictly unidirectional, we aim to investigate their relationship from both perspectives. Fourth, as outlined in the previous points, a comprehensive assessment of musicality and language—especially when multiple variables are considered—requires substantial methodological effort and resources. Given this complexity, alternative or simpler testing methods are often explored in research and practice. Self-evaluation scales represent one such approach, as previous studies have shown that individuals' self-assessments frequently correlate with their actual performance and, in some cases, even predict abilities across domains (for example, language skills predicted by singing performance or singing self-estimation). This raises the important question of whether self-evaluations might serve as a reliable and efficient alternative to more resource-intensive performance measures for assessing musical and linguistic skills.

In the present study, self-evaluation refers to participants' judgments of their own perceived musical and linguistic abilities. The individuals providing these judgments were the participants themselves, rather than experts evaluating another person's performance. The self-evaluation measures were therefore not treated as direct measures of objective competence. Instead, they were intended to capture participants' perceived competence, including general self-beliefs and retrospective evaluations of their performance on specific tasks. Objective musical and linguistic competence was assessed separately using performance-based measures. The comparison between the two types of assessment allowed us to examine the degree of correspondence and discrepancy between perceived competence and objectively measured performance.

Despite the widespread use of self-evaluation measures in both music and language research, the extent to which such measures reflect objective performance remains insufficiently understood. Self-evaluation measures are widely used across educational, psychological, and cognitive research to assess perceived competence, confidence, and self-related judgments (Ross, 1998; Zell and Krizan, 2014). In educational contexts, self-evaluations are commonly applied to assess perceived academic competence and self-regulation (Andrade, 2019; Brown and Harris, 2014), whereas cognitive research frequently employs self-report measures to investigate perceived cognitive abilities and metacognitive awareness (Zell and Krizan, 2014). Research on metacognition and self-assessment suggests that subjective evaluations of one's own abilities only partially overlap with objective performance measures, as self-evaluations may additionally reflect confidence, self-concept, metacognitive monitoring, and biases in self-perception (Zell and Krizan, 2014). In this context, self-assessment accuracy is commonly interpreted as an indicator of metacognitive monitoring processes (Nelson and Narens, 1990). Accordingly, subjective self-evaluations often diverge from objective performance, including systematic over- and underestimation of abilities (Dunning et al., 2004; Kruger and Dunning, 1999). Thus, self-evaluation studies are valuable not only for their practical applications, but also because they reveal how certain groups may systematically over- or underestimate their abilities (Groß et al., 2023). Such discrepancies may indicate theoretically meaningful differences between performance-related and self-referential aspects of cognition rather than mere measurement error.

In music and language research, self-evaluation measures are commonly used to assess perceived abilities such as musicality, singing, rhythmic skills, pronunciation, language aptitude, and confidence in learning or performance (Correia et al., 2025; Müllensiefen et al., 2014; Christiner, 2013; Christiner and Reiterer, 2013, 2015). Importantly, previous studies have shown that such subjective ratings are often associated with objective performance measures, although the strength of these associations varies substantially across individuals and domains (Correia et al., 2025; Yeom et al., 2026; Zell and Krizan, 2014). Similarly, in language research, self-evaluation items often assess perceived pronunciation ability, reading competence, language learning aptitude, verbal memory, and confidence in spoken communication (Blanche and Merino, 1989). Participants may be asked if they are good language learners, can imitate foreign accents accurately, feel confident speaking a foreign language, or can easily memorize new vocabulary (Christiner and Reiterer, 2013; Sparks et al., 2009). Educational and psycholinguistic studies also use self-reports to evaluate literacy skills, reading and writing difficulties, and confidence in oral and written production (Kaushanskaya and Marian, 2009). Importantly, self-evaluations in both domains are often associated with objective performance measures, though the strength and accuracy of these associations vary across individuals and domains (Correia et al., 2025; Yeom et al., 2026; Zell and Krizan, 2014).

Both single-item and multi-item approaches are commonly used in self-evaluation research. Whereas single-item measures are often employed for broad global estimates of competence, multi-item scales allow more differentiated assessment of perceived abilities and generally provide broader construct coverage and higher reliability (Andrade, 2019; Ross, 1998). Previous music and language research has employed both approaches to assess perceived competence, confidence, and task-related self- estimation (Correia et al., 2025; Müllensiefen et al., 2014).

To date, however, relatively little research has directly compared the relationship between subjective self-evaluation and objective performance across both music and language domains within the same experimental framework. As a result, it remains unclear whether self-evaluation operates similarly across domains, whether it can serve as a reliable proxy for performance-based assessment, and how subjective and objective measures jointly contribute to cross-domain prediction. In this study, we aimed to assess self-evaluation in both music and language performance using a multi-item approach, and to directly compare self-perceived abilities across these two domains. To obtain broad and more reliable composite measures, we included perceptual music variables—rhythm and melody perception—as well as singing ability, which together formed a composite musicality variable. For language, we used three measures that have been repeatedly associated with musicality: auditory phonological pattern recognition, speech imitation, and expressive reading ability.

To systematically capture participants' subjective assessments, we developed two multi-item self-evaluation scales—one for language and one for music—each consisting of 10 items. Although validated self-report instruments such as the Goldsmiths Musical Sophistication Index (Gold-MSI; Müllensiefen et al., 2014) for musicality are available, we opted not to adopt an existing instrument because we aimed to keep the number of items low and to use comparable, domain-specific scales for language and music within the online testing procedure. We deliberately kept the number of items low, as brief scales are commonly used in research to balance measurement breadth with participant burden. Importantly, our scales included both general items referring to typical abilities in the respective domain (e.g., “I sing the notes of melodies correctly right away”) and task-specific items directly referencing the actual tasks performed (e.g., “I sang the song I sang before very beautifully”). The item pools intentionally included both broadly framed competence judgments and items referring directly to the preceding tasks. Their empirical structure was subsequently examined using exploratory factor analysis rather than assuming a priori that these item types represented distinct constructs.

Based on these methodological considerations, the present study addressed the following research questions. First, we examined whether performance in one domain (music or language) could be predicted by both performance and self-evaluation measures from the other domain (Q1 and Q2). Second, we explored the joint predictive value of performance and self-evaluation measures from both domains for each outcome, that is, whether language performance can be predicted by music performance, music self-evaluation, and language self-evaluation, and vice versa for music performance (Q3 and Q4). In this context, both music and language performance were treated as dependent variables, reflecting the possibility of bidirectional associations between the two domains (Q5). Finally, we aimed to assess the degree of agreement between subjective self-evaluations and objective performance measures in both domains, in order to determine whether self-evaluations could serve as reliable alternatives to more resource-intensive performance-based assessments (Q6).

## 2 Materials and methods

### 2.1 Participants

A total of 104 participants (67 female and 37 male) were included in the study (age: M = 33.29, SD = 11.34). All participants provided informed consent and took part voluntarily. Recruitment was conducted via online platforms and university-based channels. Inclusion criteria required participants to be at least 18 years of age. Additionally, participants were required not to speak Mandarin or Tagalog, as this was essential to ensure equal opportunities to perform well in speech imitation and auditory phonological pattern recognition tasks.

Demographic information, including age, gender, and musical background, was collected via self-report (see Supplementary Table S16). For descriptive purposes, participants reported the average number of hours per week they spent singing and playing an instrument over the past 3 years. The mean number of hours spent singing was 1.90 (SD = 2.79, range = 0–16), and the mean number of hours spent playing an instrument was 1.70 (SD = 2.96, range = 0–15).

### 2.2 Language measures

The present study focuses on three related but distinct language-related abilities: auditory phonological pattern recognition, speech imitation, and expressive reading. While each task targets a different aspect of language processing and production, all are theoretically linked to musicality and have been associated with musical perception and skills in previous research (Christiner, 2020; Groß et al., 2023; Christiner and Groß, 2025; Christiner et al., 2026; Groß and Christiner, 2026; Christiner and Reiterer, 2013, 2018; Christiner, 2018). Although the three language measures were only weakly intercorrelated, we used a composite language score in the main analyses to obtain a pragmatic summary index of overall language performance. To assess the robustness of the language score as a composite measure, we additionally conducted sensitivity analyses using each language measure separately. These analyses, reported in the Supplementary materials (see Sections 2 and 3), indicated a broadly consistent pattern across the three language measures: the effects were in the same direction, although one effect was weaker and did not reach conventional significance, while remaining positive. Accordingly, the main manuscript reports analyses based on the language summary index as the primary outcome measure.

#### 2.2.1 Expressive reading

We assessed expressive reading ability using a paradigm in which participants read aloud the German version of the fable “The North Wind and the Sun,” following the procedure established by Christiner et al. (2026). Participants were instructed to deliver the text in a manner that would engage and sustain the attention of adult listeners, thus promoting an expressive and audience-oriented reading style.

All recordings were inspected and processed prior to evaluation. This included checking audio quality and normalizing loudness levels to ensure comparability across participants. The recordings were then uploaded to an internally developed, purpose-built web-based platform, which was specifically designed to facilitate the online assessment of speech and singing recordings using study-specific rating interfaces (the same procedure was followed for the speech imitation and singing tasks described in the subsequent sections). Performance was evaluated using expert ratings by trained raters. Raters were required to meet two predefined criteria: they were native speakers of German and had a linguistic background. To ensure consistency in evaluation, raters were provided with the same instructions as the participants and were asked to judge how effectively each reading captured and sustained listener engagement throughout the narrative.

Ratings were provided on a continuous scale ranging from 0 (“low”) to 10 (“high”), reflecting the perceived degree of expressive and engaging delivery. A minimum of three raters per task was ensured to obtain reliable ICC estimates; larger numbers of raters further increase score precision and data robustness (Koo and Li, 2016). This principle was applied consistently across all tasks involving rating procedures reported in the later sections. Interrater reliability was assessed using intraclass correlation coefficients (ICCs), which exceeded the commonly accepted threshold of 0.70, indicating satisfactory agreement among raters (see Supplementary Table 1).

#### 2.2.2 Speech imitation

Speech imitation ability was assessed using a set of stimuli previously validated in research on individual differences in language (Christiner et al., 2022a, 2023, 2021, 2022b,c). The task measures participants' capacity to encode and reproduce unfamiliar phonological material with minimal prior exposure. This assessment has high ecological validity, as it simulates the early stages of foreign language learning, during which individuals typically imitate new words or phrases. Stimuli consisted of short utterances ranging from nine to eleven syllables, produced by six native speakers (three female, three male) of Tagalog and Mandarin. Both languages were selected due to their low familiarity among German native speakers, thereby minimizing potential effects of prior linguistic exposure.

Each stimulus was presented auditorily three times, separated by brief pauses of 300 ms. Following presentation, participants were instructed to reproduce the utterances as accurately as possible. A familiarization phase preceded the main task to ensure procedural understanding, during which participants were exposed to Tagalog stimuli not included in the experimental set. The main task comprised six target items from both languages, presented in randomized order.

All recordings were checked for quality, trimmed where necessary, and normalized for loudness prior to evaluation. Performance was assessed by native speakers of the respective target languages using a purpose-built web-based rating platform (Mandarin: *n* = 40; Tagalog: *n* = 16). Raters evaluated overall imitation accuracy on a continuous scale ranging from 0 to 10. During the rating procedure, raters first listened to the original recording and were subsequently presented with the participant's imitation. They were asked to evaluate, on a global level, how accurately the participant had imitated the original recording. Raters were informed that the participants had neither previously spoken nor been taught the target language and that some utterances might therefore not be fully intelligible. They were also instructed to focus on the overall similarity between the two recordings rather than on the semantic comprehensibility of the utterance.

Interrater reliability was assessed using intraclass correlation coefficients (ICCs), which exceeded the conventional threshold of 0.70, indicating satisfactory agreement across raters (see Supplementary Tables 2, 3).

In line with previous findings indicating that typological differences between languages do not substantially influence immediate imitation performance (Christiner et al., 2021, 2022a), ratings from both language conditions were aggregated. The resulting composite score serves as a global index of participants' capacity to reproduce unfamiliar speech material upon first exposure.

#### 2.2.3 Auditory phonological pattern recognition

Auditory phonological pattern recognition was assessed using a shortened version of a previously validated task (Christiner et al., 2026), designed to capture the ability to identify, segment, and recognize unfamiliar phonological sequences based on their acoustic and prosodic characteristics. The stimulus material was derived from recordings of *The North Wind and the Sun* and produced by native speakers across a large set of languages.

Tagalog and Mandarin were used as stimulus languages due to their low familiarity among German native speakers, thereby minimizing potential effects of prior linguistic exposure while preserving natural phonetic and prosodic variability. The material was recorded by six native speakers per language (three female, three male). Stimuli consisted of words and short phrases ranging from three to eleven syllables and were normalized for loudness.

Each trial followed a two-part structure comprising a stimulus sequence (“string”) and a subsequent probe (“response stimulus”). The string consisted of multiple segments presented in succession with brief silent intervals of approximately 300 ms. Depending on the condition, strings varied in length (e.g., eight, ten, or twelve segments) and systematically sampled different levels of phonological complexity by combining shorter (three to six syllables) and longer (seven to eleven syllables) units.

Following the presentation of the string, a delay of 2 s was introduced, indicated by a change in screen color, before the response stimulus was presented. Response stimuli consisted of one to three segments. Participants were required to indicate whether all elements of the response stimulus had occurred within the preceding string. Responses were scored as correct if all probe elements were present in the string, irrespective of order.

To maintain consistency of prosodic cues, all segments within a given trial were produced by the same speaker. Participants completed 12 trials per language, with items randomly drawn from different string lengths and balanced with respect to match and non-match conditions. Performance was quantified as mean accuracy across trials. Previous large-scale validations have demonstrated approximately normal score distributions (Christiner, 2020; Christiner et al., 2026; Christiner and Groß, 2025; Coumel et al., 2023).

#### 2.2.4 Language self-evaluation

Language self-evaluation was measured using a multi-item self-report scale designed to capture participants' perceived language competence across a range of relevant domains. The scale comprised ten items, each rated on an 11-point Likert scale from 0 (“not at all”) to 10 (“completely”). The items were developed to reflect both general language ability and specific skills corresponding to the three performance tasks administered in the study: speech imitation, expressive reading, and auditory phonological pattern recognition. For example, participants were asked to evaluate their ease with speech listening tasks, their ability to imitate languages, and their reading proficiency, as well as their overall confidence and perceived talent in language learning and task performance.

The item pool was constructed to ensure coverage of both the specific skills targeted by the objective performance tasks and more general aspects of language self-concept. This approach was intended to strike a balance between task-specific self-assessment and broader evaluations of language competence, thereby providing a comprehensive measure of perceived language ability.

To examine the structure of the self-evaluation measures, we conducted an exploratory factor analysis of the 10 items. One item was excluded because of its low communality (*h*^2^ = 0.13). The subsequent EFA conducted on the remaining nine items yielded a two-factor solution, with all communalities exceeding *h*^2^ = 0.20. Based on the item content, the factors were interpreted as representing a general language ability dimension and a more task-specific dimension. The general language ability factor, comprising six items, showed good internal consistency (Cronbach's α= 0.79; McDonald's ω = 0.80), whereas the three-item task-specific factor showed borderline internal consistency (Cronbach's α = 0.66; McDonald's ω = 0.70). Consequently, only the general language ability factor was retained for the subsequent analyses. Detailed information on the factor-analytic procedure and the reliability analyses is provided in Section 4 of the Supplementary materials, which also lists the six statements retained for the final analysis.

### 2.3 Music

Musicality is widely conceptualized as a multidimensional construct encompassing a range of distinct but related musical abilities. To comprehensively assess musical ability, we included four established performance-based musicality tasks: singing a familiar song (“Happy Birthday”), the Beat Alignment Test (BAT), the Melodic Discrimination Task (MDT), and the Mistuning Perception Task (MPT). Each task targets a different fundamental aspect of musical ability—singing ability, rhythm perception, melodic discrimination, and meter perception, respectively. In contrast to the language measures, the musicality measures are more closely intercorrelated (see Supplementary materials. Section 5). Composite measures derived from multiple assessments are therefore commonly used to provide a broader estimate of overall musicality by integrating information across complementary domains of musical ability (Harrison et al., 2017; Law and Zentner, 2012). We expected the musicality measures to be closely interrelated. To examine their associations and underlying factor structure, we conducted correlational analyses and an exploratory factor analysis (EFA; see Supplementary materials, Section 6). The results showed moderate-to-strong intercorrelations among the musicality measures, and the EFA yielded a single-factor solution. All four variables loaded substantially on this factor: Singing Happy Birthday (0.736), BAT ability (0.608), MDT ability (0.786), and MPT ability (0.666). The four musicality measures were z-transformed and averaged to create a unit-weighted composite score of musical ability. In all subsequent analyses, only this composite score was used as the measure of musical ability, rather than the individual task scores.

#### 2.3.1 Singing

Singing ability was assessed using a standardized performance task based on the well-established “*Happy Birthday”* paradigm, which has been shown to be suitable for individuals with varying levels of musical expertise (Christiner and Reiterer, 2013; Christiner et al., 2022a,b, 2026). Participants were instructed to sing a short excerpt of the song as accurately as possible using their natural singing voice.

All recordings were inspected and preprocessed prior to evaluation, including the removal of prolonged pre-phonatory silence and normalization of loudness levels to ensure comparability across participants. The processed recordings were then uploaded to a web-based rating platform.

Performance was evaluated by a panel of nine professional musicians, who rated each recording along four predefined dimensions: melodic accuracy, rhythmic precision, vocal range, and voice quality. Ratings were provided on a 0–10 scale, anchored at 0 (“low”) and 10 (“high”). For each participant, the four singing-task ratings were averaged to obtain a composite singing performance score, with higher scores indicating better perceived singing performance. This scoring procedure followed established procedures (Christiner et al., 2026). Interrater reliability was assessed using intraclass correlation coefficients (ICCs), which indicated moderate to good agreement among raters (ICC range: 0.70–0.88; see Supplementary Table 4).

#### 2.3.2 Mistuning perception test (MPT)

Sensitivity to vocal pitch deviations was assessed using the Mistuning Perception Test (MPT; Larrouy-Maestri et al., 2019), an adaptive listening paradigm designed to quantify individual differences in the detection of pitch inaccuracies in sung music.

Stimuli consisted of short audio excerpts (~6–12 s) derived from commercially available pop recordings. For each excerpt, the vocal track was either presented in its original form or digitally altered by shifting its pitch relative to the accompanying instrumental track.

Each trial followed a two-alternative forced-choice (2AFC) format. Participants were presented with two versions of the same musical excerpt in randomized order—one unaltered and one containing a pitch-manipulated vocal line—and were required to identify the version in which the singing was out of tune.

The test employed an adaptive procedure in which item difficulty was continuously adjusted based on participants' responses, allowing efficient and precise estimation of mistuning sensitivity. Previous validation studies have demonstrated satisfactory psychometric properties, including good test–retest reliability and evidence for convergent and divergent validity (Larrouy-Maestri et al., 2019).

#### 2.3.3 Melodic discrimination ability (MDT)

Melodic discrimination ability was assessed using the Melodic Discrimination Test (MDT; Harrison et al., 2017)), an adaptive, computer-based measure designed to capture individual differences in the detection of local pitch deviations within melodic sequences.

Each trial followed a three-alternative forced-choice (3-AFC) format. Participants were presented with three short melodic sequences, two of which were structurally identical apart from a global transposition, while the third contained a localized pitch deviation that altered the melodic contour. Participants were required to identify the deviant sequence.

Stimuli were generated algorithmically based on a statistical model derived from a corpus of Irish folk melodies. The sequences ranged from 3 to 16 notes and varied systematically in difficulty as a function of structural properties such as contour integrity, tonal predictability, and melodic expectancy. Global transpositions were applied to prevent reliance on absolute pitch, thereby isolating relative pitch processing.

The test was administered via the psychTestR platform and employed an adaptive procedure in which item selection was adjusted in real time based on participant responses. Previous validation studies have demonstrated good psychometric properties, including high internal consistency (Cronbach's α > 0.80) and evidence for construct validity (Harrison et al., 2017).

#### 2.3.4 Computerized adaptive beat alignment test (CA-BAT)

Beat perception ability was assessed using the Computerized Adaptive Beat Alignment Test (CA-BAT; Harrison and Müllensiefen, 2018), a performance-based measure designed to quantify sensitivity to temporal alignment in musical contexts.

Each trial followed a two-alternative forced-choice (2-AFC) format. Participants listened to two versions of the same short musical excerpt (~5 s), each superimposed with a sequence of metronome-like beeps. In one version, the beeps were temporally aligned with the underlying musical beat, whereas in the other they were systematically displaced. Participants were required to identify the version in which the beeps were better synchronized with the music.

Stimuli were drawn from a set of 32 musical excerpts spanning different genres, with reference beat positions established based on expert drummer annotations. The test employed an adaptive procedure in which item difficulty was adjusted in real time as a function of participants' responses, allowing efficient and precise estimation of beat perception ability.

Previous validation studies have demonstrated satisfactory psychometric properties, including evidence for construct validity through associations with musical training (*r* ≈ 0.40) and acceptable test–retest reliability under controlled conditions (*r* = 0.67; Harrison and Müllensiefen, 2018).

#### 2.3.5 Musical self-evaluation

Musical self-evaluation was measured using a multi-item self-report scale designed to assess participants' perceived musical ability across a range of relevant domains. The scale comprised ten items, each rated on an 11-point Likert scale from 0 (“not at all”) to 10 (“completely”). The items were developed to reflect both general musical competence and specific skills corresponding to the four performance tasks administered in the study: singing (“Happy Birthday”), melodic pattern test (MPT), melodic discrimination test (MDT), and the the Computerized Adaptive Beat Alignment Test (CA-BAT). For example, participants were asked to evaluate their ability to sing melodies accurately, their sense of rhythm, their capacity to distinguish and remember melodies, and their overall confidence in solving musical tasks.

Analogous to the language self-evaluation scale, the item pool was constructed to ensure coverage of both the specific skills targeted by the objective performance tasks and more general aspects of musical self-concept. This approach was intended to strike a balance between task-specific self-assessment and broader evaluations of musical competence, thereby providing a comprehensive measure of perceived musical ability. To examine the underlying structure of these items, we conducted an exploratory factor analysis (EFA) on all 10 self-evaluation items. Although our scale included both general and task-specific items, the EFA revealed a single-factor solution, indicating that all items measured a common underlying construct. The internal consistency of the scale was excellent (Cronbach's α = 0.95, McDonald's ω = 0.95; a detailed approach is provided in the Supplementary material, section 7).

### 2.4 Experimental setting and procedure

All participants were assessed individually in a controlled laboratory environment. The auditory tasks were administered using the same laboratory computers and a standardized external audio interface. Sound was presented binaurally through closed dynamic Sennheiser HDA 200 headphones. Before data collection, the auditory output was calibrated to a sound-pressure level of 65 dB. Participants could not independently modify the volume during the experimental tasks. This standardized setup reduced variability attributable to differences in playback equipment, ambient sound, or individually selected listening levels.

Spoken, read, and sung responses were recorded in the same laboratory environment using an identical recording configuration. Participants received standardized instructions for each task and completed the designated practice or familiarization trials before beginning the corresponding experimental trials.

The measures were administered in two consecutive domain-specific blocks. In the language block, participants first completed an expressive reading of the German version of “The North Wind and the Sun,” followed by the speech imitation task and the auditory phonological pattern recognition task. They subsequently completed the Language Competence Self-Evaluation scale.

In the music block, participants first sang an excerpt from “Happy Birthday” and then completed the Mistuning Perception Test (MPT), the Melodic Discrimination Test (MDT), and the Computerized Adaptive Beat Alignment Test (CA-BAT). The Music Competence Self-Evaluation scale was administered after completion of these musical performance tasks. Trial- or stimulus-level randomization within individual tasks was implemented as described in the corresponding task subsections, whereas the order of the task blocks was fixed.

The self-evaluation scales were therefore administered after the corresponding objective performance tasks. This sequence was necessary because several task-specific items explicitly referred to tasks that participants had just completed, whereas the remaining items assessed their more general perceived competence in the respective domain. Participants did not receive feedback about their objective performance before completing either self-evaluation scale. Both scales used an 11-point response format ranging from 0 (“not at all”) to 10 (“completely”).

### 2.5 Statistical analysis

All statistical analyses were conducted using SPSS (version 31). The analysis proceeded as follows: first, we report descriptive statistics, followed by correlation analyses. The correlational analysis served as a screening step. As expected, all four main variables were significantly correlated with each other. Specifically, we conducted two hierarchical regression analyses, each comprising two steps, to examine cross-domain and domain-specific effects: (1) whether language performance could be predicted by music performance and/or music self-evaluation, (2) whether music performance could be predicted by language performance and/or language self-evaluation, (3) whether language performance could be predicted by music performance, music self-evaluation, and language self-evaluation, and (4) whether music performance could be predicted by language performance, language self-evaluation, and music self-evaluation. In addition, Bland–Altman plots were used to examine the agreement between subjective self-evaluations and objective performance measures. The primary aim of this analysis was to assess the extent to which self-evaluations corresponded to, or diverged from, objective performance.

## 3 Results

### 3.1 Descriptive statistics

Descriptive statistics for all variables are presented in Table 1. The Language Summary Index comprises speech imitation, expressive reading, and auditory phonological pattern recognition tasks, whereas the music performance score includes the MPT, MDT, BAT, and singing assessments. Descriptive statistics for the individual variables are provided in Supplementary material, section 8.

**Table 1:** Descriptive statistics for language and music variables.

Variable	N	Mean	SD
Language summary index	104	−0.01	0.66
Language self-evaluation	104	5.00	1.76
Music performance score	104	0.00	0.79
Music self-evaluation	104	5.06	2.17

This table presents the descriptive statistics for the main variables under consideration. The Language Summary Index comprises speech imitation, expressive reading, and auditory phonological pattern recognition tasks. The music performance score includes the MPT, MDT, BAT, and singing assessments. Means and standard deviations are reported for each variable, providing an overview of the sample characteristics.

### 3.2 Correlation analysis

Pearson correlations among all study variables are presented in Table 2. All variables were significantly correlated with one another.

**Table 2:** Correlations between language and music variables.

Variable	1	2	3	4
1. Language summary index	—			
2. Language self-evaluation	0.44^**^	—		
3. Music performance	0.44^**^	0.31^**^	—	
4. Music self-evaluation	0.21^*^	0.33^**^	0.60^**^	—

^*^p < 0.05.

^**^p < 0.01.

This table presents the correlations among the main variables. Correlation coefficients are reported, reflecting the strength and direction of the relationships between language performance, music performance, and the self-estimation variables for music and language. As anticipated, all variables show significant correlations with each other.

### 3.3 Regression analyses: cross-domain and domain-specific predictors of language and music performance

#### 3.3.1 Regression 1: predicting language performance from music domain predictors and language competence self-evaluation

A hierarchical multiple regression was conducted to examine whether *Music Total Performance Score* and *Music Competence Self-evaluation* predicted the *Language Summary Index* in Step 1, and whether *Language Competence Self-evaluation* explained additional variance in Step 2. In Step 1, the model was significant, *F*_(2, 101)_ = 12.85, *p* < 0.001, and accounted for 20.3% of the variance in language performance (*R*^2^ = 0.203, adjusted *R*^2^ = 0.187). Within this model, the *Music Total Performance Score* was a significant positive predictor of the *Language Summary Index*. Adding *Language Competence Self-evaluation* in Step 2 significantly improved the model, Δ*R*^2^ = 0.112, *F*_(1, 100)_ = 16.27, *p* < 0.001. The final model was significant, *F*_(3, 100)_ = 15.29, *p* < 0.001, and explained 31.4% of the variance in the language summary index (*R*^2^ = 0.314, adjusted *R*^2^ = 0.294). *Music Total Performance Score* remained a significant positive predictor in the final model, as did *Language Competence Self-evaluation*. *Music Competence Self-evaluation* was not a significant predictor (see Table 3 for model-fit statistics and Table 4 for regression coefficients). Variance inflation factors were low (1.15–1.62), suggesting no multicollinearity concerns, and standardized residuals ranged from −2.89 to 2.60. Visual inspection suggested that the standardized residuals were approximately normally distributed in the final hierarchical regression model, with no substantial deviations from normality (see Supplementary materials, Section 9, Supplementary Figure 1).

**Table 3:** Model fit of the hierarchical regression predicting the *Language Summary Index*.

Step	Predictors entered	*R* ^2^	Adjusted *R*^2^	Δ*R*^2^	ΔF	p for ΔF
1	Music performance; music self-evaluation	0.203	0.187	—	12.853	< 0.001
2	Language self-evaluation	0.314	0.294	0.112	16.274	< 0.001

Step 1: Music Total Performance Score and Music Competence Self-evaluation. Step 2: Language Competence Self-evaluation. The final model was significant, *F*_(3, 100)_ = 15.289, *p* < 0.001.

**Table 4:** Regression coefficients of the hierarchical regression predicting the *Language Summary Index*.

Step	Predictor	B	SE	β	*t*	*p*	95% CI for B/VIF
1	Music total performance score	0.419	0.093	0.500	4.501	< 0.001	[0.234, 0.604]; 1.564
1	Music competence self-evaluation	−0.061	0.073	−0.093	−0.837	0.405	[−0.207, 0.084]; 1.564
2	Music total performance score	0.366	0.088	0.437	4.168	< 0.001	[0.192, 0.540]; 1.600
2	Music competence self-evaluation	−0.115	0.070	−0.174	−1.652	0.102	[−0.253, 0.023]; 1.623
2	Language competence self-evaluation	0.236	0.059	0.358	4.034	< 0.001	[0.120, 0.352]; 1.151
	Constant (step 1/step 2)	−0.010/−0.010	0.058/0.054	—	−0.165/−0.175	0.869/0.861	[−0.125, 0.106]/[−0.117, 0.098]

B, unstandardized coefficient; SE, standard error; β, standardized coefficient; CI, confidence interval; VIF, variance inflation factor. VIF values are reported for the corresponding model. The dependent variable was the Language Summary Index.

#### 3.3.2 Regression 2: predicting music performance from language domain predictors and music competence self-evaluation

A hierarchical multiple regression was conducted to examine whether *Language Summary Index* and *Language Competence Self-evaluation* predicted music performance, and whether *Music Competence Self-evaluation* explained additional variance in music performance beyond these predictors. *Music Total Performance Score* was entered as the dependent variable. In Step 1, the *Language Summary Index* and *Language Competence Self-evaluation* were entered simultaneously. The model was significant, *F*_(2, 101)_ = 13.83, *p* < 0.001, and explained 21.5% of the variance in music performance (*R*^2^ = 0.215, adjusted *R*^2^ = 0.199). Within this model, the *Language Summary Index* was a significant positive predictor of music performance.

In Step 2, *Music Competence Self-evaluation* was added to the model. This addition significantly improved the model, Δ*R*^2^ =0.253, *F*_(1, 100)_ = 47.46, *p* < 0.001. The final model was significant, *F*_(3, 100)_ = 29.28, *p* < 0.001, and accounted for 46.8% of the variance in *Music Total Performance Score* (*R*^2^ = 0.468, adjusted *R*^2^ = 0.452). In the final model, the *Language Summary Index* remained a significant positive predictor of music performance. *Music Competence Self-evaluation* was also a significant positive predictor. In contrast, *Language Competence Self-evaluation* was not significantly associated with music performance in the final model (see Table 5 for model-fit statistics and Table 6 for regression coefficients). VIF values ranged from 1.131 to 1.338, indicating no apparent multicollinearity concerns. Standardized residuals ranged from −2.660 to 2.096, and the residual histogram did not indicate substantial deviations from normality (see Supplementary materials, Section 9, Supplementary Figure 2).

**Table 5:** Model fit of the hierarchical regression predicting the *Music Total Performance Score*.

Step	Predictors entered	*R* ^2^	Adjusted *R*^2^	Δ*R*^2^	ΔF	p for ΔF
1	Language summary index; language self-evaluation	0.215	0.199	—	13.826	< 0.001
2	Music self-evaluation	0.468	0.452	0.253	47.455	< 0.001

Step 1: Language Summary Index and Language Competence Self-evaluation. Step 2: Music Competence Self-evaluation. The final model was significant, *F*_(3, 100)_ = 29.275, *p* < 0.001.

**Table 6:** Regression coefficients of the hierarchical regression predicting the *Music Total Performance Score*.

Step	Predictor	B	SE	β	t	p	95% CI for B/VIF
1	Language summary index	0.453	0.117	0.380	3.875	< 0.001	[0.221, 0.685]; 1.236
1	Language competence self-evaluation	0.116	0.077	0.147	1.504	0.136	[−0.037, 0.269]; 1.236
2	Language summary index	0.404	0.097	0.339	4.168	< 0.001	[0.212, 0.597]; 1.243
2	Language competence self-evaluation	−0.010	0.066	−0.013	−0.154	0.878	[−0.142, 0.121]; 1.338
2	Music competence self-evaluation	0.420	0.061	0.535	6.889	< 0.001	[0.299, 0.542]; 1.131
	Constant (step 1/step 2)	0.006/0.006	0.069/0.057	—	0.087/0.097	0.931/0.923	[−0.131, 0.143]/[−0.108, 0.119]

B, unstandardized coefficient; SE, standard error; β, standardized coefficient; CI, confidence interval; VIF, variance inflation factor. VIF values are reported for the corresponding model.

### 3.4 Bland-Altman plots

Details are provided below in Figures 1 and 2.

**Figure 1:**
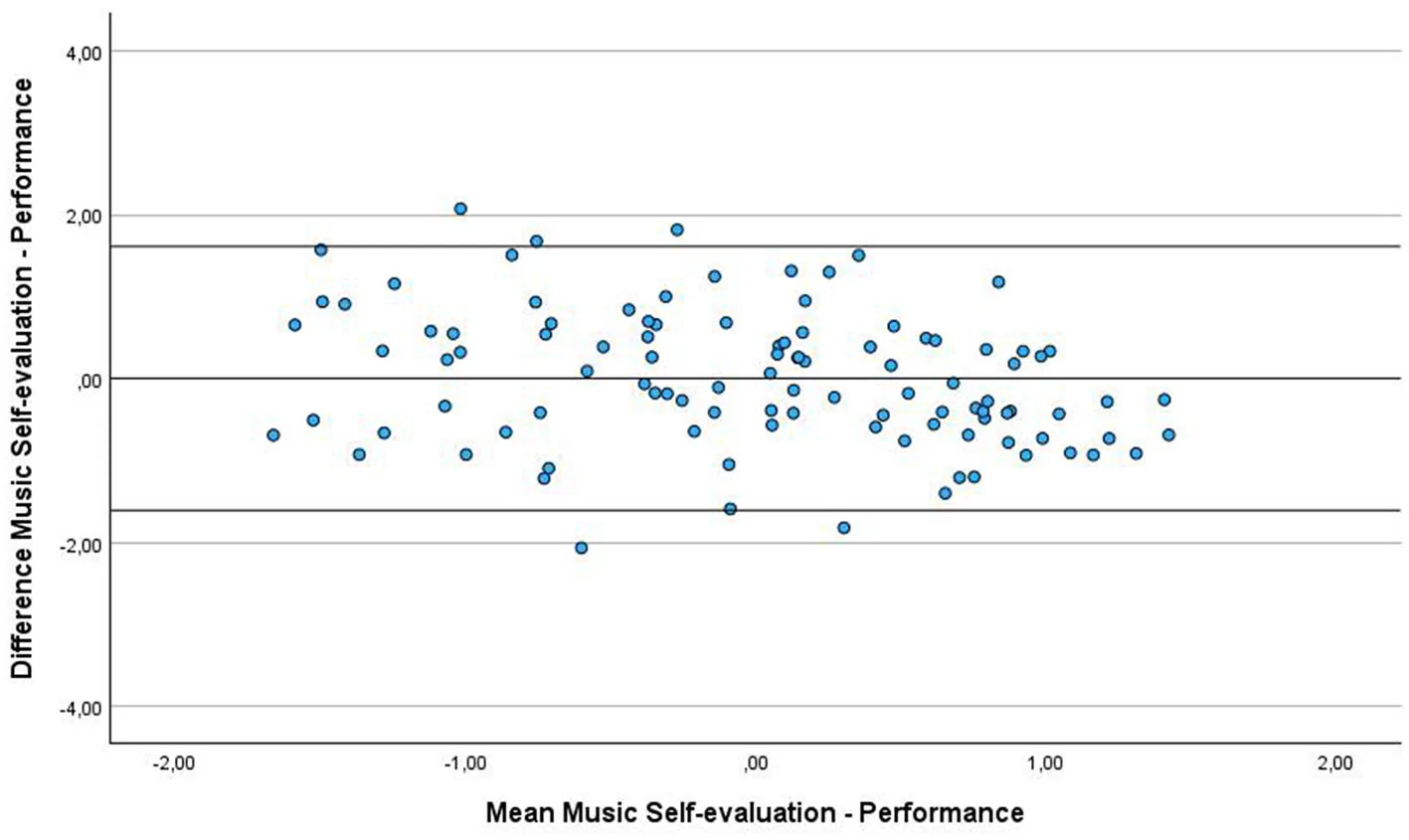
Bland-Altman plot shows the agreement between subjective self-evaluation and objective performance in music. The plot illustrates the individual differences (over- and underestimation) between self-assessed and actual performance scores.

**Figure 2:**
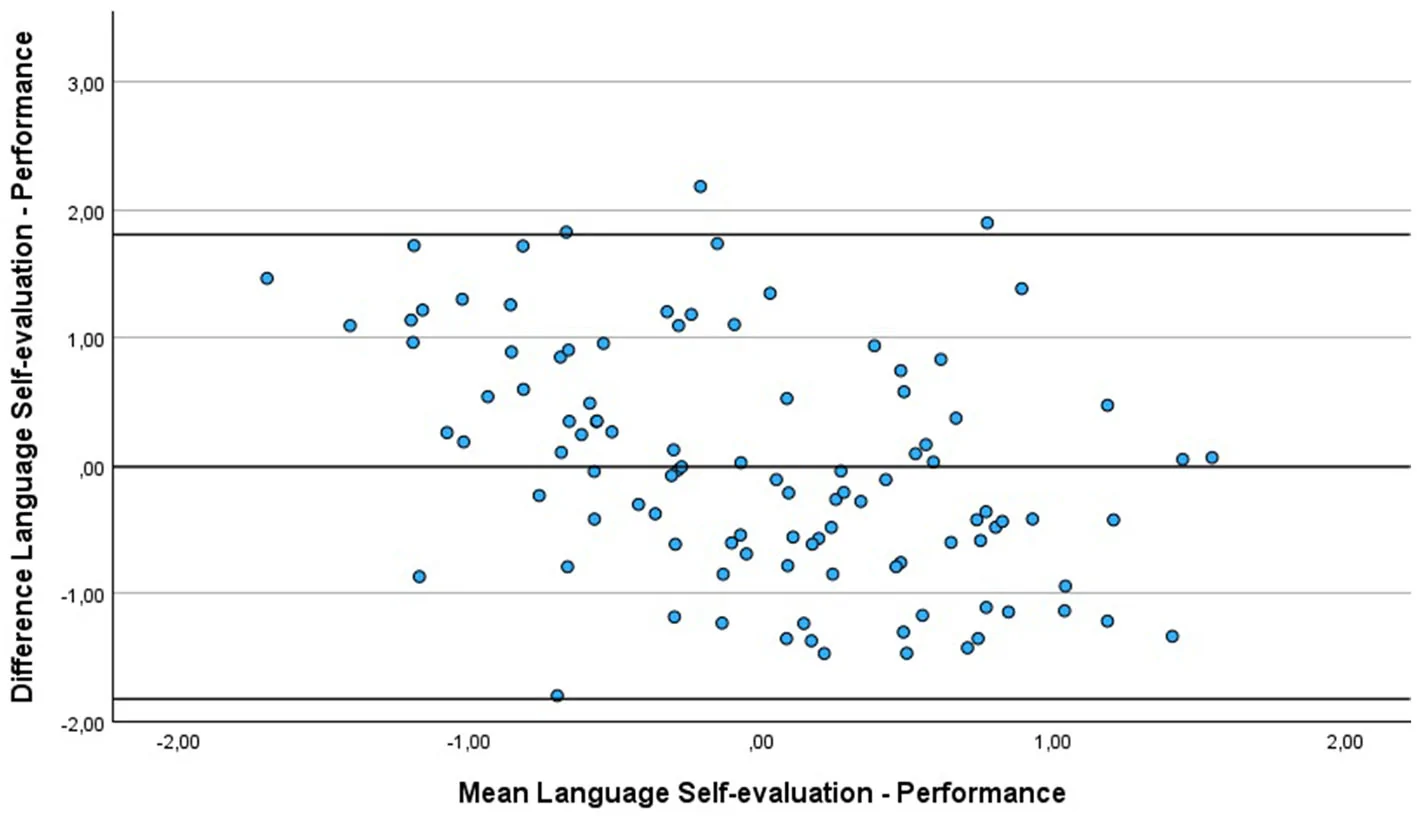
Bland-Altman plot shows the agreement between subjective self-evaluation and objective performance in the language domain. The solid line represents the mean difference (bias), and the dashed lines indicate the 95% limits of agreement.

#### 3.4.1 Bland-Altman plots: music performance score and music self-evaluation

The Bland-Altman analysis revealed a mean difference of 0.002 between subjective self-evaluation and objective performance in the music domain, with 95% limits of agreement ranging from approximately −1.6 to +1.6 (see Figure 1). The differences were broadly dispersed, indicating substantial individual variability in the agreement between self-assessed and actual performance. The negligible mean difference suggested no meaningful systematic bias at the group level.

#### 3.4.2 Bland-Altman plots: language summary index and language self-evaluation

The Bland-Altman analysis for the language domain revealed a mean difference of −0.009 between subjective self-evaluation and objective performance, with a standard deviation of 0.92. The 95% limits of agreement ranged from ~-1.82 to +1.81 (see Figure 2). The differences were widely distributed, indicating substantial individual variability in the agreement between self-assessed and actual language performance. Although the mean difference indicated no meaningful systematic bias at the group level, the wide limits of agreement suggested considerable individual variability in the correspondence between self-evaluations and objective language performance.

## 4 Discussion

At the outset, we addressed several research questions. First (Q1 & Q2), we examined whether performance in one domain (music or language) could be predicted by both performance and self-evaluation measures from the other domain. Second (Q3 & Q4), we explored the combined predictive value of performance and self-evaluation from both domains for each performance outcome. Third (Q6), we assessed the degree of agreement between subjective self-evaluations and objective performance outcomes to determine whether self-evaluations could serve as reliable alternatives to performance-based assessments. Finally (Q5), we considered the possibility of bidirectional associations by treating both music and language performance as dependent variables.

Our findings revealed moderate associations between subjective self-evaluation and objective performance in both music and language domains. When predicting performance across domains, objective performance measures proved to be stronger predictors than self-evaluation. Associations between music and language performance were largely bidirectional, supporting the interpretation of partially shared cross-domain mechanisms, although the strength of these associations varied depending on the specific measures used. Finally, while self-evaluation measures provided some insight into participants' abilities, they did not consistently serve as reliable substitutes for objective performance assessments.

Based on these findings, we will discuss (1) the relationship between self-evaluation and objective performance within and across domains, (2) the impact on the use of self-evaluation measures, and (3) the bidirectional associations between music and language abilities.

### 4.1 Interpretation of the self-evaluation measures

Our results revealed positive associations between self-evaluation and objective performance in both domains, with a correlation of *r* = 0.45 for language and *r* = 0.60 for music. These findings are consistent with evidence that individuals generally have moderate, but incomplete, insight into their abilities (Zell and Krizan, 2014). However, the difference between the two correlations should be interpreted cautiously because the factor analyses indicated that the musical and language self-evaluation scores did not represent fully equivalent constructs.

For musical self-evaluation, all ten items loaded on a common factor, and the complete scale showed excellent internal consistency (Cronbach's α = 0.95; McDonald's ω = 0.95). The data therefore did not support a psychometric separation between the items addressing general musical competence and those referring to the musical tasks completed in the study. The Music Competence Self-Evaluation score used in the subsequent analyses should consequently be interpreted as an integrated measure of perceived musical competence that combines general musical self-beliefs with retrospective evaluations of the tasks performed during the assessment.

The one-factor solution does not demonstrate that global and task-specific musical evaluations are theoretically identical. It indicates that these item types were not empirically distinguishable with the present item pool and sample. This qualification is consistent with research showing that self-reported musical sophistication can be represented by a general factor while also comprising more specific facets of musical behavior and perceived ability (Müllensiefen et al., 2014). Thus, the current findings support the use of a combined score in the present analyses but do not exclude the possibility that a more balanced or extensive item set would reveal distinct global and task-specific dimensions.

A different structure emerged for language self-evaluation. After one item with low communality had been removed, the remaining items formed two correlated factors, interpreted as General Language Ability and Task-Specific Competence. The six-item General Language Ability factor showed good internal consistency (Cronbach's α = 0.79; McDonald's ω = 0.80). The Task-Specific Competence factor comprised only three items and showed lower, borderline internal consistency (Cronbach's α = 0.66; McDonald's ω = 0.70). It was therefore not retained in the main analyses. Accordingly, the Language Competence Self-Evaluation score used in the regression models represents general perceived language ability rather than participants' evaluations of their performance on the specific language tasks.

This difference in scale composition limits direct comparisons between musical and language self-evaluation. The music analyses relate objective musical performance to a combined scale containing both global and task-related content, whereas the language analyses relate objective language performance exclusively to global self-evaluation. Previous research likewise indicates that correspondence between perceived and objectively assessed musical ability can vary substantially; for example, Wise and Sloboda (2008) demonstrated that self-evaluation of singing ability does not necessarily map closely onto objective performance. The higher correlation observed in the music domain may therefore reflect not only differences between musical and language self-evaluation but also differences in construct composition, item wording and content, and correspondence between the self-evaluation items and the performance criteria. Research in second-language assessment similarly indicates that the validity of self-evaluation varies across language skills and with learners' experience of the activity being evaluated (Ross, 1998). The present analyses cannot determine whether a sufficiently reliable task-specific language scale would have shown a stronger or weaker association with objective language performance.

The hierarchical regression models nevertheless showed a consistent within-domain contribution of self-evaluation. Language Competence Self-Evaluation explained additional variance in the Language Summary Index after the music-domain predictors had been entered, whereas Music Competence Self-Evaluation explained additional variance in the Music Total Performance Score after the language-domain predictors had been entered. In contrast, the respective cross-domain self-evaluation measure did not make a significant unique contribution in the final model. This pattern supports the domain relevance of self-evaluation but should not be taken as evidence that the musical and language self-evaluation coefficients are directly comparable, given the different composition of the two scales.

The timing of the task-specific items further qualifies their interpretation. Because these items referred retrospectively to tasks that participants had already completed, they may have been influenced by immediate perceptions of task difficulty and success. Such scale-level evaluations differ from trial-by-trial confidence judgments used to quantify metacognitive sensitivity, which require confidence ratings to be linked directly to the accuracy of individual responses (Fleming and Lau, 2014). The present self-evaluation scales should therefore be interpreted as measures of perceived competence rather than direct measures of objective ability or metacognitive monitoring accuracy. Their associations with performance indicate partial correspondence between perceived and objectively measured competence, but do not establish that the two forms of assessment are interchangeable.

The Bland–Altman plots complement these findings by illustrating substantial interindividual variation in over- and underestimation. They do not identify the psychological source of these discrepancies or explain the regression coefficients. Rather, they show that similar standardized self-evaluation and performance scores at the group level can coexist with substantial individual disagreement, further indicating that self-evaluation cannot be treated as interchangeable with objective assessment.

Future studies should use larger and equally balanced sets of global and task-specific items in both domains and evaluate their structure in an independent sample using confirmatory factor analysis. General competence judgments could be collected before the performance tasks, whereas task-specific evaluations could be obtained immediately after each corresponding task. If the aim is to assess metacognitive monitoring more directly, confidence ratings should additionally be collected at the trial level and analyzed in relation to response accuracy. Such a design would permit a clearer examination of whether general self-beliefs and task-specific evaluations contribute differently to musical and language performance.

A particularly relevant consideration in this field is the prevailing research asymmetry: most studies have focused on the influence of musical abilities on language, whereas the potential contribution of linguistic abilities to musical performance has received considerably less empirical attention. This imbalance is theoretically difficult to justify for several reasons. One reason is that converging neuroscientific evidence points to partially shared processing mechanisms, grounded in structural similarities in the acoustic signals of speech and music (Ding et al., 2017; Schön et al., 2004). Accordingly, a bidirectional relationship appears plausible, a notion further supported by findings that tone-language speakers exhibit enhanced pitch-related musical abilities compared to non-tone-language speakers, suggesting that language experience itself may shape musical performance (Bidelman et al., 2013). Another reason supporting the plausibility of a bidirectional relationship is that several studies have suggested that associations between music and language may not solely reflect transfer effects from musical training, but may also arise from shared domain-general mechanisms or stable individual differences influencing performance in both domains (Swaminathan and Schellenberg, 2020; Kragness et al., 2021).

In the present study, comparable association patterns emerged irrespective of whether music or language was treated as the outcome variable. Musical abilities predicted language performance, and linguistic abilities predicted music performance. Although these findings do not permit conclusions about causal directionality, they suggest that associations between music and language can be meaningfully examined from both perspectives. Rather than supporting a strictly unidirectional transfer account, the present findings are consistent with the assumption that music and language partly rely on overlapping perceptual, cognitive, and auditory processing mechanisms. Consequently, the observed associations could be interpreted as reflecting a combination of shared underlying capacities and domain-specific experiences rather than a simple transfer from one domain to the other.

## 5 Limitations

Several limitations should be considered when interpreting the present findings. First, the order of the two domain-specific task blocks was fixed rather than counterbalanced, with the language block preceding the music block. Although trial- or stimulus-level randomization was implemented within individual tasks where applicable, potential order, fatigue, practice, or carry-over effects therefore cannot be excluded. The self-evaluation scales were administered after the corresponding performance tasks because several items explicitly referred to the tasks participants had just completed. Nevertheless, future studies should counterbalance the order of the two domains to disentangle potential order effects from domain-specific associations. Second, the music and language domains were operationalized using different task formats and showed different psychometric structures. The musical performance measures were moderately to strongly intercorrelated and supported a single-factor solution, whereas the language measures were only weakly intercorrelated and were therefore summarized using a pragmatic Language Summary Index, supplemented by sensitivity analyses of the individual language tasks. Consequently, the two domain-level performance scores should not be regarded as strictly equivalent psychometric constructs, and direct comparisons between music and language should be interpreted with appropriate caution. Third, formal musical training and broader musical background were not examined as potential covariates or moderators. These factors may influence both objective musical performance and the calibration of self-evaluations, particularly in the musical domain, and may therefore contribute to individual differences in the observed associations. Future research should address these limitations by using counterbalanced task orders, more closely matched cross-domain measures, and larger samples that permit explicit modeling of training- and experience-related factors.

## 6 Conclusion

Our findings demonstrate that subjective self-evaluation and objective performance are moderately associated within and across music and language domains yet represent partially dissociable constructs. Objective performance measures consistently showed stronger associations with cross-domain outcomes than self-evaluation measures, suggesting that subjective ratings cannot fully substitute for performance-based assessment. At the same time, self-evaluation contributed additional explanatory variance in some models, indicating that subjective ratings may capture aspects beyond objectively measured performance, including confidence-related or self-referential components. Cross-domain analyses further revealed comparable association patterns irrespective of whether music or language served as the outcome variable, suggesting that similar mechanisms may operate across both domains. However, the musical and language self-evaluation measures were not psychometrically equivalent: the musical score combined global and task-specific content, whereas the language analyses were based exclusively on the global factor. Direct comparisons between the predictive contributions of the two self-evaluation measures should therefore be made cautiously. Overall, the present findings highlight the importance of integrating subjective self-evaluation and objective performance measures when investigating individual differences in music and language, as both approaches appear to provide complementary but non-interchangeable information.

## Data Availability

The raw data supporting the conclusions of this article will be made available by the corresponding author upon reasonable request.
