# Supplementary material for: Trusting self-evaluation: predicting music and language performance from subjective ratings
Source: Front Psychol. 2026 Sep 21;17:1899488. doi: 10.3389/fpsyg.2026.1899488 (PMC13634953; doi:10.3389/fpsyg.2026.1899488)

Supplementary Information

This Supplementary Information provides additional psychometric and statistical analyses supporting the main manuscript. Tables S1–S4 report intraclass correlation coefficients for the rating-based performance measures. Tables S5–S8 present correlations among the language measures and sensitivity analyses of the Language Summary Index. Tables S9–S12 document the factor structure and internal consistency of the Language Competence Self-Evaluation scale. Tables S13–S15 report correlations among the musical performance measures and the factor-analytic and reliability results for the musical performance and self-evaluation measures. Table S16 shows participant demographics and musical background characteristics. Table S17 provides descriptive statistics for the individual study variables. Figures S1–S2 show the standardized residual distributions of the two regression models, and Figures S3–S6 present the distributions of the four principal study variables.

1. Intraclass correlation coefficients

Tables S1–S4 present the intraclass correlation coefficients (ICCs) for the rating-based performance measures, including expressive reading, speech imitation, and singing performance. These coefficients quantify the level of agreement among raters and provide an index of the reliability and consistency of the evaluations across participants. Intraclass correlation coefficients (ICCs) were calculated using a two-way mixed-effects model with absolute agreement, with participants treated as random effects and measurement occasions/raters treated as fixed effects. The tables report average-measures ICCs, reflecting the reliability of the mean rating across all raters. Intraclass correlation coefficients (ICCs) for average measures exceeding 0.70 are generally considered indicative of good interrater reliability. In general, a minimum of three raters is required to obtain reliable ICCs; in our study, we consistently used a larger number of raters, as increasing the number of raters enhances score precision.

Table S1. Intraclass correlation coefficients: Expressive Reading

	**Intraclass Correlation**	**95% Confidence Interval**		**F Test with True Value 0**			
		Lower Bound	*Upper Bound*	*Value*	*df1*	*df2*	*Sig*
**Average Measures**	.73	.62	.81	4.16	99	396	< .001

Table S2. Intraclass correlation coefficients: Speech Imitation – Tagalog Sentences

Tagalog Sentence	Intraclass Correlation	95% Confidence Interval		F Test with True Value 0			
		Lower Bound	*Upper Bound*	*Value*	*df1*	*df2*	*Sig*
Sentence 1	.92	.89	.95	17.72	100	1500	< .001
Sentence 2	.93	.91	.95	18.49	101	1515	< .001
Sentence 3	.93	.90	.95	22.11	101	1515	< .001
Sentence 4	.94	.92	.96	22.26	102	1530	< .001
Sentence 5	.93	.90	.95	18.20	103	1545	< .001
Sentence 6	.93	.90	.95	18.71	103	1545	< .001

Note. Intraclass correlation coefficients (ICCs) represent *average measures* based on a two-way mixed-effects model with absolute agreement.

Table S3. Intraclass correlation coefficients: Speech Imitation – Mandarin Sentences

Mandarin Sentence	Intraclass Correlation	95% Confidence Interval		F Test with True Value 0			
		Lower Bound	*Upper Bound*	*Value*	*df1*	*df2*	*Sig*
Sentence 1	.96	.95	.97	38.20	101	3939	< .001
Sentence 2	.98	.97	.98	49.82	99	3861	< .001
Sentence 3	.97	.96	.98	45.09	101	3939	< .001
Sentence 4	.97	.96	.98	48.01	99	3861	< .001

**Mandarin Sentence**	**Intraclass Correlation**	**95% Confidence Interval**		**F Test with True Value 0**			
Sentence 5	.96	.94	.97	37.17	100	3900	< .001
Sentence 6	.96	.95	.97	40.12	100	3900	< .001

Note. Intraclass correlation coefficients (ICCs) represent *average measures* based on a two-way mixed-effects model with absolute agreement.

Table S4. Intraclass correlation coefficients: Singing Happy Birthday

**Variable**	**Intraclass Correlation**	**95% Confidence Interval**		**F Test with True Value 0**			
		Lower Bound	*Upper Bound*	*Value*	*df1*	*df2*	*Sig*
Melody	.86	.77	.91	12.50	102	816	< .001
Rhythm	.70	.48	.80	6.83	102	816	< .001
Vocal range	.88	.79	.92	14.73	102	816	< .001
Quality of voice	.80	.67	.88	10.36	102	816	< .001

Note. Intraclass correlation coefficients (ICCs) represent *average measures* based on a two-way mixed-effects model with absolute agreement.

2. **Spearman correlations among language measures and language self-evaluation**

Table S5 below shows the correlations between the language variables and participants' self-evaluation of their language ability. The results show that, as stated in the Introduction, the language variables are only weakly correlated.

Table S5. Spearman correlations

Variable	1	2	3	4
1. North Wind and the Sun Reading	—			
2. Speech Imitation	0.177*	—		
3. Auditory Phonological Pattern Recognition	0.089	0.167*	—	
4. Language Competence Self-evaluation	0.368**	0.331**	0.194*	—

Note. Spearman correlations among language measures. Values below the diagonal. ** p < .01 (1-tailed); * p < .05 (1-tailed).

3. **Language summary index**

Although the three language measures were only weakly intercorrelated, we used a composite language score (Language Summary Index) in the main analyses. Prior research has shown that all three measures are related to musicality, and the composite score offers a more comprehensive and robust representation of language performance than any single language predictor alone. To evaluate the robustness of the Language Summary Index, we also conducted sensitivity analyses in which the individual language measures were examined separately. In separate models, North Wind and the Sun Reading, Speech Imitation, and Auditory Phonological Pattern Recognition were regressed on Language Competence Self-evaluation, Music Total Performance Score, and Music Competence Self-evaluation (all z-standardized). All variables were z-standardized prior to analysis. This sensitivity analysis examined whether the pattern observed for the Language Summary Index also held for the individual language tasks. The results were broadly consistent across models. Overall, the results of the individual regression models were broadly in line with the pattern observed for the language summary index, with comparable positive effects of language self-evaluation and musical performance on expressive reading and speech imitation, and weaker, non-significant but directionally similar effects for auditory phonological pattern recognition. The results of the three regression models are reported below.

3.1 Regression model predicting North Wind and the Sun Reading

A multiple regression analysis was conducted to examine whether language competence self-evaluation, music competence self-evaluation, and music performance predicted North Wind and the Sun Reading. The model was significant, $F(3, 100) = 7.45$, $p < .001$, and accounted for 18.9% of the variance in language performance (see table S6 below). All variance inflation

factors (VIFs) ranged from 1.16 to 1.74, indicating no concerns regarding multicollinearity. The standardized residuals ranged from -2.38 to 2.41, suggesting the absence of extreme outliers.

Table S6. Sensitivity analysis: Multiple regression predicting North Wind and the Sun Reading

**Predictor**	**B**	**SE**	**β**	**t**	**p**	95% CI for B
Constant	-0.897	0.298	–	-3.005	.003	[-1.489, -0.304]
Music Total Performance Score	0.362	0.152	.283	2.385	.019	[0.061, 0.664]
Language Competence Self-evaluation	0.179	0.056	.314	3.166	.002	[0.067, 0.291]
Music Competence Self-evaluation	-0.089	0.119	-.090	-0.747	.457	[-0.325, 0.147]

3.2 Regression model predicting Speech Imitation

A multiple regression analysis was conducted to examine whether language competence self-evaluation, music competence self-evaluation, and music performance predicted *Speech Imitation*. The model was significant, $F(3, 99) = 8.7$, $p < .001$, and accounted for 19.6% of the variance in language performance (see table S7 below). All variance inflation factors (VIFs) ranged from 1.15 to 1.72, indicating no concerns regarding multicollinearity. The standardized residuals ranged from -2.16 to 2.71, suggesting the absence of extreme outliers.

Table S7. Sensitivity analysis: Multiple regression predicting Speech Imitation

**Predictor**	**B**	**SE**	**β**	**t**	**p**	95% CI for B
Constant	-0.712	0.288	–	-2.472	.015	[-1.283, -0.141]
Music Total Performance Score	0.486	0.149	.377	3.255	.002	[0.190, 0.782]
Language Competence Self-evaluation	0.143	0.055	.252	2.610	.010	[0.034, 0.251]
Music Competence Self-evaluation	-0.123	0.117	-.124	-1.047	.297	[-0.356, 0.110]

3.3 Regression model predicting Auditory Phonological Pattern Recognition

A multiple regression analysis was conducted to examine whether language competence self-evaluation, music competence self-evaluation, and music performance predicted *Auditory Phonological Pattern Recognition*. The model was non-significant, $F(3, 99) = 2.02$, $p = .116$, and accounted for 6% of the variance in language performance (see table S8 below). All variance inflation factors (VIFs) ranged from 1.13 to 1.76, indicating no concerns regarding

multicollinearity. The standardized residuals ranged from -2.95 to 1.81, suggesting the absence of extreme outliers.

Table S8. Sensitivity analysis: Multiple regression predicting Auditory Phonological Pattern Recognition

Predictor	B	SE	β	t	p	95% CI for B
Constant	-0.489	0.322	–	-1.520	.132	[-1.128, 0.150]
Music Total Performance Score	0.300	0.168	.228	1.788	.077	[-0.033, 0.632]
Language Competence Self-evaluation	0.095	0.061	.164	1.559	.122	[-0.026, 0.216]
Music Competence Self-evaluation	-0.169	0.133	-.167	-1.273	.206	[-0.432, 0.094]

4. Language Competence Self-Evaluation Scale

We conducted an exploratory factor analysis (EFA) on all 10 self-evaluation items to examine the underlying factor structure. One item with a low communality value ($h^2 = .13$) was excluded from further analyses. A second EFA on the remaining nine items revealed a two-factor solution, with all items showing communalities above $h^2 = .20$. Each factor was interpreted based on item content and labelled accordingly. Internal consistency for both subscales was assessed using Cronbach's alpha and McDonald's omega. Factor 1 represented a more general language assessment construct comprising six items and showed good internal consistency (Cronbach's $\alpha = .79$; McDonald's $\omega = .80$), whereas Factor 2 comprised three task-specific items and showed borderline internal consistency (Cronbach's $\alpha = .66$; McDonald's $\omega = .70$). Therefore, only Factor 1 was used in the subsequent analyses. The full statistical approach to the analysis of the multi-item scales is reported in the subsequent sections.

4.1 Language Competence Self-Evaluation Items

The items of the Language Competence Self-Evaluation Scale are presented below.

a. The speech listening tasks which I performed before are easy.
b. Imitating languages such as pronunciation exercises in foreign languages I do very well.
c. I would estimate my reading ability as very good.
d. I have very good potential to learn languages.
e. I have no difficulties when I am asked to solve language tasks.
f. I am a language talent and perform well on any language task
g. I feel confident when I have to remember and retain words from unfamiliar languages.

h. How would you assess your foreign language proficiency?
i. I am confident in my ability to learn new foreign languages.
j. I feel confident reading in my native and foreign languages.

4.2 Exploratory Factor Analysis (Principal Axis Factoring, Oblimin Rotation)

An exploratory factor analysis (principal axis factoring, Oblimin rotation) was conducted on the 10 language self-evaluation items. The Kaiser–Meyer–Olkin measure indicated acceptable sampling adequacy (KMO = .74), and Bartlett's test of sphericity was significant, $\chi^2(45) = 392.66$, $p < .001$. Two correlated factors emerged, explaining 41.7% of the variance (extracted eigenvalues: 3.52 and 0.65). The first factor captured general language potential and ability (e.g., "I have very good potential to learn languages", "I am a language talent and perform well on any language task"), whereas the second factor reflected task-specific ease and competence (e.g., speech listening, pronunciation, reading). The item "I feel confident when I have to remember and retain words from unfamiliar languages" showed a low communality ($h^2 = .13$) and was therefore excluded from further analyses. However, the factor correlation was moderate ($r = .43$), indicating that both factors share a common core of positive language self-evaluation. Table S9 shows the factor loadings and communalities.

Table S9 Factor Loadings for Language Self-Evaluation Items

Note. Principal axis factoring, Oblimin rotation. Table shows pattern matrix loadings for the two correlated factors (F1, F2) and extracted communalities (h^2).

Item	F1 loading	F2 loading	Communality (h^2)
The speech listening tasks which I performed before are easy.	-0.05	0.75	0.53
Imitating languages such as pronunciation exercises in foreign languages I do very well.	0.21	0.51	0.39
I would estimate my reading ability as very good.	-0.08	0.57	0.29
I have very good potential to learn languages.	0.65	0.36	0.75

Item	F1 loading	F2 loading	Communality (h^2)
I have no difficulties when I am asked to solve language tasks.	0.41	0.46	0.55
I am a language talent and perform well on any language task.	0.70	0.32	0.79
I feel confident when I have to remember and retain words from unfamiliar languages.	0.20	0.22	0.13
How would you assess your foreign language proficiency?	0.39	0.12	0.21
I am confident in my ability to learn new foreign languages.	0.58	-0.13	0.29
I feel confident reading in my native and foreign languages.	0.53	-0.07	0.25

4.3 EFA of the Remaining Nine Items

A second exploratory factor analysis (principal axis factoring, Oblimin rotation) was conducted on the 9 language self-evaluation items. The Kaiser–Meyer–Olkin measure indicated acceptable sampling adequacy (KMO = .74), and Bartlett's test of sphericity was significant, $\chi^2(36) = 389.05$, $p < .001$. Two correlated factors emerged, explaining 57.4% of the variance (extracted eigenvalues: 3.47 and 0.7). The first factor captured general language potential and ability (e.g., "I have very good potential to learn languages"; "I am a language talent and perform well on any language task"), whereas the second factor reflected task-specific ease and competence (e.g., speech listening, pronunciation, reading). The correlation between the two factors was moderate ($r = .47$), indicating that both factors share a common core of positive language self-evaluation. Although some items showed relatively low communalities, they were retained in the

analysis due to their theoretical and content relevance. Table S10 shows the factor loadings and communalities.

Table S10 Factor Loadings for Language Self-Evaluation Items after removal of the item "I feel confident when I have to remember and retain words from unfamiliar languages".

Item	F1 loading	F2 loading	Communality (h^2)
The speech listening tasks which I performed before are easy.	-0.12	0.88	0.68
Imitating languages such as pronunciation exercises in foreign languages I do very well.	0.21	0.53	0.43
I would estimate my reading ability as very good.	0.05	0.45	0.22
I have very good potential to learn languages.	0.76	0.22	0.79
I have no difficulties when I am asked to solve language tasks.	0.48	0.37	0.54
I am a language talent and perform well on any language task.	0.80	0.19	0.83
How would you assess your foreign language proficiency?	0.42	0.09	0.22
I am confident in my ability to learn new foreign languages.	0.50	-0.07	0.22
I feel confident reading in my native and foreign languages.	0.52	-0.09	0.23

4.4 Self-Evaluation Items Assigned to the Two Identified Factors

Table S11 presents the items from the self-evaluation scales grouped according to the two factors identified in the factor analysis: General Language Ability and Task-Specific Competence.

**Factor 1 (General Language Ability)**	**Factor 2 (Task-Specific Competence)**
I have very good potential to learn languages.	The speech listening tasks which I performed before are easy.
I am a language talent and perform well on any language task.	Imitating languages such as pronunciation exercises in foreign languages I do very well.
I have no difficulties when I am asked to solve language tasks.	I would estimate my reading ability as very good.
How would you assess your foreign language proficiency?	
I am confident in my ability to learn new foreign languages.	
I feel confident reading in my native and foreign languages.	

Note: The item "I have no difficulties when I am asked to solve language tasks." showed a primary loading of 0.48 and a secondary loading of 0.37 (difference = 0.11). The item was retained in the 6-item scale. This crossloading is reported for transparency.

4.5 Internal Consistency (Cronbach's Alpha and Omega) Factor 1

The internal consistency of the 6-item factor was assessed using Cronbach's Alpha and McDonald's Omega. The results indicated good reliability (Cronbach's Alpha = .79; McDonald's Omega = .80). All items showed acceptable corrected item-total correlations (ranging from .42 to .76), and the removal of any item did not substantially increase reliability. These findings suggest that the scale demonstrates good internal consistency (see Table S12). We calculated a mean score for the self-evaluation scale by averaging the responses to the six items. The general language ability factor was subsequently used in the main analyses reported in the manuscript.

Table S12. Item-total statistics and reliability coefficients for the 6-item Language Competence Self-Evaluation scale.

Item	Cronbach's Alpha if Item Deleted	McDonald's Omega if Item Deleted
I have very good potential to learn languages.	.718	.710
I am a language talent and perform well on any language task	.710	.706
I have no difficulties when I am asked to solve language tasks.	.755	.751
How would you assess your foreign language proficiency?	.788	.780
I am confident in my ability to learn new foreign languages.	.792	.813
I feel confident reading in my native and foreign languages.	.799	.821

4.6 Internal Consistency (Cronbach's Alpha and Omega) Factor 2

The internal consistency of the 3-item factor was assessed using Cronbach's Alpha (.66) and McDonald's Omega (.70). Corrected item-total correlations ranged from .36 to .57. Item means ranged from 3.38 to 6.77 (SDs 2.25–2.46). These findings indicate borderline reliability and should be interpreted with caution. Given the borderline reliability and limited number of items, we do not include the 3-item scale in subsequent analyses and focus exclusively on the 6-item concept.

5. Spearman Correlations Among Musical Performance Measures

Table S13 shows the correlations among the musical performance measures.

Variable	1	2	3	4
1. Singing Happy Birthday	—			
2. BAT ability	0.481**	—		
3. MDT ability	0.614**	0.488**	—	
4. MPT ability	0.486**	0.358**	0.497**	—

Note. Spearman correlations among musical ability measures. ** p < .01 (1-tailed); * p < .05 (1-tailed).

6. Exploratory Factor Analysis of Musical Performance Measures Using Principal Axis Factoring

A principal axis factor analysis was conducted on the four musical performance variables: Singing Happy Birthday, BAT ability, MDT ability, and MPT ability. The data were suitable for factor analysis, with a Kaiser-Meyer-Olkin measure of sampling adequacy of .788 and Bartlett's test of sphericity being significant, $\chi^2(6) = 113.23$, $p < .001$. The variables showed moderate intercorrelations, ranging from .404 to .578.

The analysis yielded a single factor with an eigenvalue of 1.97, explaining 49.33% of the variance. All four variables loaded substantially on this factor: Singing Happy Birthday (.736), BAT ability (.608), MDT ability (.786), and MPT ability (.666). Communalities ranged from .370 to .618. The reproduced correlations closely matched the observed correlations, and no residuals exceeded .05. Because only one factor was extracted, rotation was not applicable.

7. Musical Competence Self-Evaluation Scale

To examine the underlying structure of these items, we conducted an exploratory factor analysis (EFA) on all 10 self-evaluation items. Although our scale included both general and task-specific items, the EFA revealed a single-factor solution, indicating that all items measured a common underlying construct. The items of the Musical Competence Self-Evaluation Scale are presented below. The items were each rated on an 11-point Likert scale ranging from 0 (strongly disagree/not at all) to 10 (strongly agree/completely).

a. I sing the notes of melodies correctly right away.
b. I sang the song I sang before very beautifully.
c. Rhythm changes are difficult to hear. But I think I can do it very well.
d. I have a very good sense of rhythm.
e. Distinguishing melodies is very easy for me.
f. I have no difficulty remembering and retaining melodies.
g. Musicality tasks like I did before are not difficult to solve.
h. I hear when music or music parts sound wrong.
i. I think I did very well on the music tasks I did before.
j. I can tell if someone is hitting the notes.

7.1 Exploratory Factor Analysis of Musical Competence Self-Evaluation Using Principal Axis Factoring

A principal axis factor analysis was conducted on the ten self-reported musical ability items. The data were suitable for factor analysis, with a Kaiser–Meyer–Olkin measure of sampling adequacy of .911 and Bartlett's test of sphericity being significant, $\chi^2(45) = 961.00$, $p < .001$. The variables showed moderate to strong intercorrelations, ranging from .349 to .880 (all ps < .001). Principal axis factor analysis using the Kaiser criterion (eigenvalues > 1) yielded a single factor with an eigenvalue of 6.86, explaining 68.57% of the total variance. All items

loaded substantially on this factor, with loadings ranging from .59 to .93. Communalities (squared loadings) ranged from .35 to .86 (see also Table S14).

Table S14. Factor Loadings for Musical Competence Self-Evaluation Items

Note. Principal axis factoring, one-factor solution. Table S14 shows standardized factor loadings and extracted communalities (h^2) for the Musical Competence Self-Evaluation items.

Item	Factor Loading	Communality (h^2)
I sing the notes of melodies correctly right away.	0.774	0.600
I sang the song I sang before very beautifully.	0.591	0.350
Rhythm changes are difficult to hear. But I think I can do it very well.	0.876	0.768
I have a very good sense of rhythm.	0.844	0.712
Distinguishing melodies is very easy for me.	0.849	0.721
I have no difficulty remembering and retaining melodies.	0.670	0.449
Musicality tasks like I did before are not difficult to solve.	0.751	0.564
I hear when music or music parts sound wrong.	0.929	0.864
I think I did very well on the music tasks I did before.	0.841	0.708
I can tell if someone is hitting the notes.	0.900	0.810

7.2 Internal Consistency of the Musical Competence Self-Evaluation Scale

The internal consistency of the scale was excellent (Cronbach's $\alpha = .95$, McDonald's $\omega = .95$). Table S15 shows Cronbach's Alpha and McDonald's Omega if each item was deleted.

Table S15. Reliability Analysis: Cronbach's Alpha and McDonald's Omega

**Item**	**Cronbach's Alpha if item deleted**	**McDonald's Omega if item deleted**
I sing the notes of melodies correctly right away.	.940	.942
I sang the song I sang before very beautifully.	.949	.949

Item	Cronbach's Alpha if item deleted	McDonald's Omega if item deleted
Rhythm changes are difficult to hear. But I think I can do it very well.	.937	.937
I have a very good sense of rhythm.	.938	.938
Distinguishing melodies is very easy for me.	.938	.938
I have no difficulty remembering and retaining melodies.	.945	.944
Musicality tasks like I did before are not difficult to solve.	.942	.942
I hear when music or music parts sound wrong.	.934	.934
I think I did very well on the music tasks I did before.	.938	.938
I can tell if someone is hitting the notes.	.935	.934

8. Participant Demographics and Musical Background Characteristics

Table S16. Participant Demographics and Musical Background Characteristics

Variable	Statistic	Value
Age	M (SD)	33.29 (11.34)
Female participants	n (%)	67 (64.4%)
Male participants	n (%)	37 (35.6%)
Singing experience	Hours/week, M (SD)	1.90 (2.79)
Instrument-playing experience	Hours/week, M (SD)	1.70 (2.96)
Total sample	N	104

Note. Singing and instrument-playing values refer to the average number of hours per week reported for the previous three years.

8.1 Descriptive Statistics for Individual Variables

Descriptive statistics for all individual variables are presented in Table S17.

Table S17. Descriptive Statistics for Individual Study Variables

Variable	Median	Std. Deviation	Minimum	Maximum
North Wind and the Sun Reading	5.4000	1.24013	1.80	8.40

Variable	Median	Std. Deviation	Minimum	Maximum
Speech Imitation	8.2972	1.58372	5.27	12.62
Auditory Phonological Pattern Recognition	9.0000	1.38120	5.00	11.00
Language Competence Self-evaluation	5.3333	1.77668	0.67	8.67
Singing Happy Birthday	6.1389	1.53694	1.64	8.61
BAT ability	0.03429859819	0.9122539949	-2.583501869	1.708459284
MDT ability	-0.08426213301	0.9124777284	-2.597439574	1.705335030
MPT ability	0.3383086297	0.8753081631	-2.092430256	1.766999037
Music Competence Self-evaluation	5.1000	2.12645	0.60	8.90

9. Histograms of Standardized Residuals of the Two Regression Models

Figure S1

Histogram of Standardized Residuals for the Language Summary Index Model

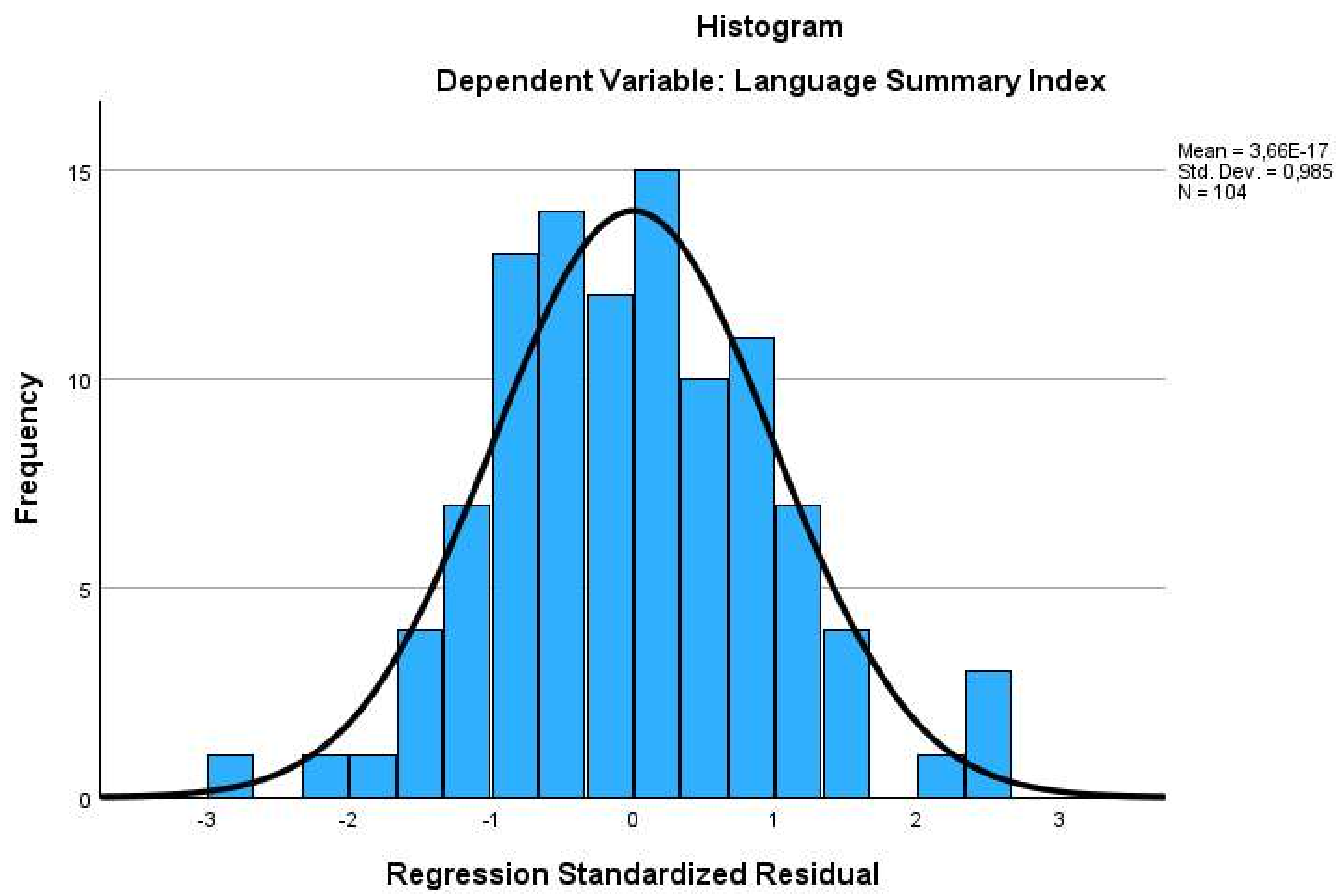

Figure S2

Histogram of Standardized Residuals for the Music Total Performance Score Model

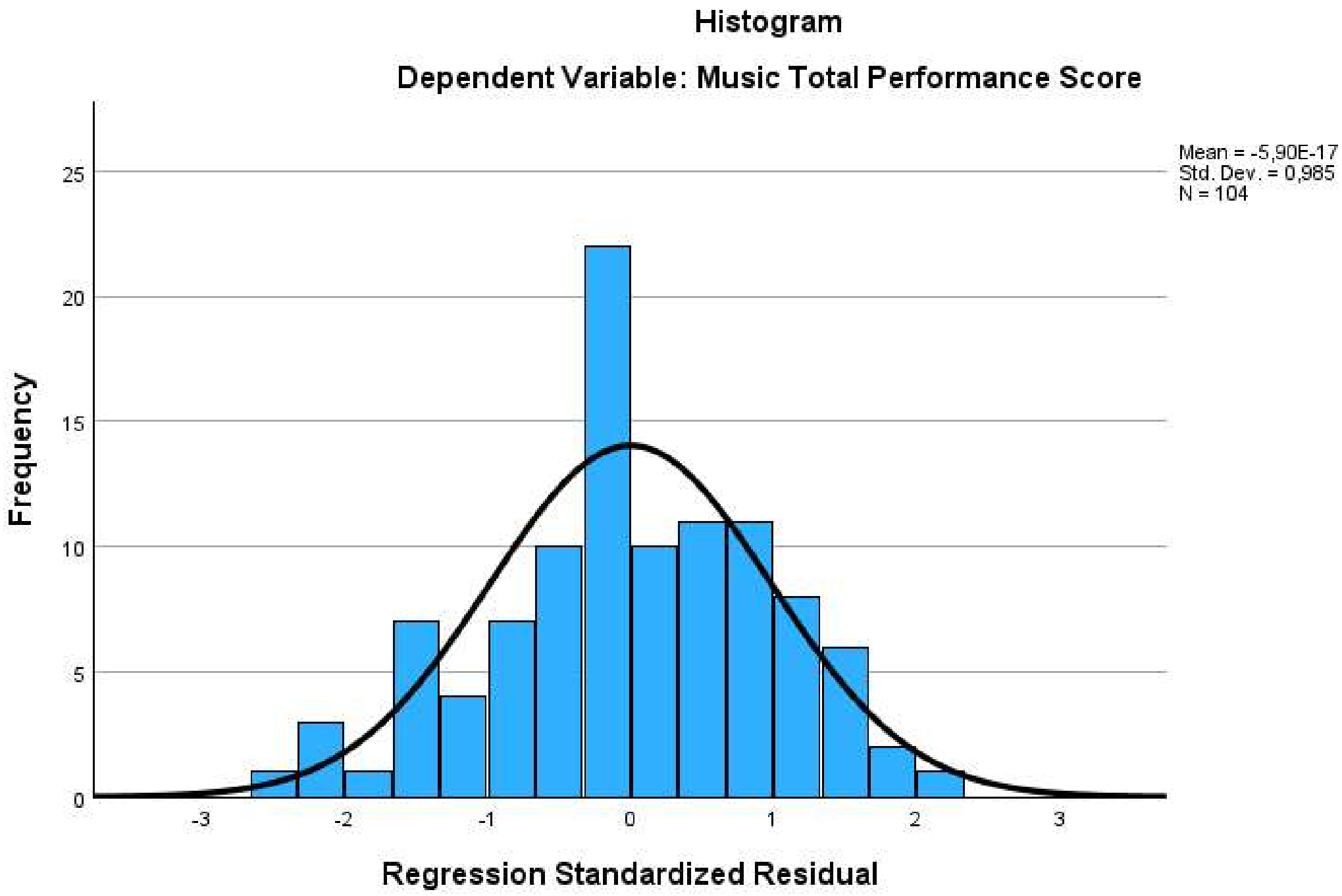

10. Distributions of the Four Main Study Variables

Figures S3–S6 present histograms of the four primary study variables and provide a visual overview of the distribution of scores in the sample.

Figure S3. Distribution of the Music Total Performance Score in the sample.

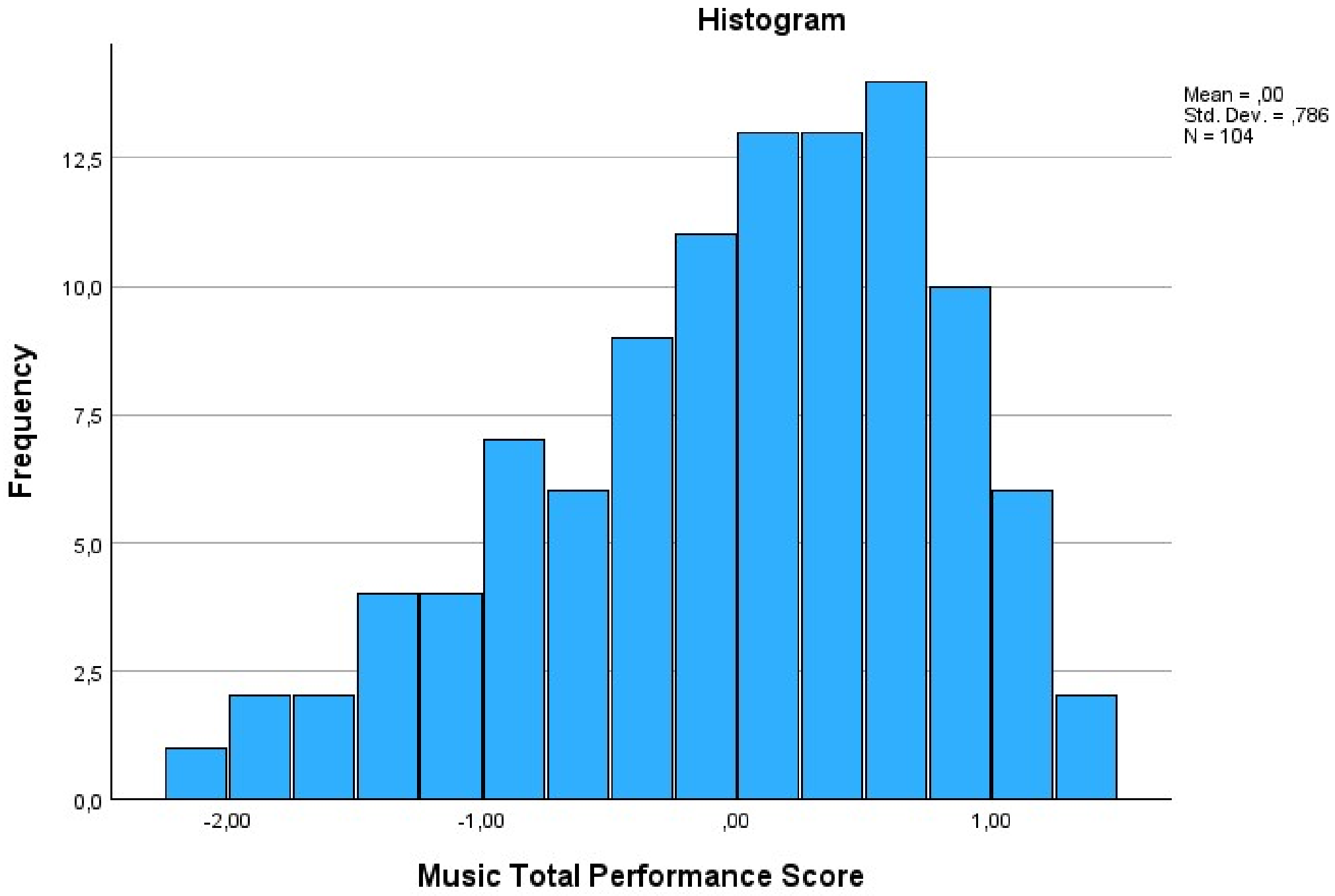

Figure S4. Distribution of the Music Competence Self-evaluation in the sample.

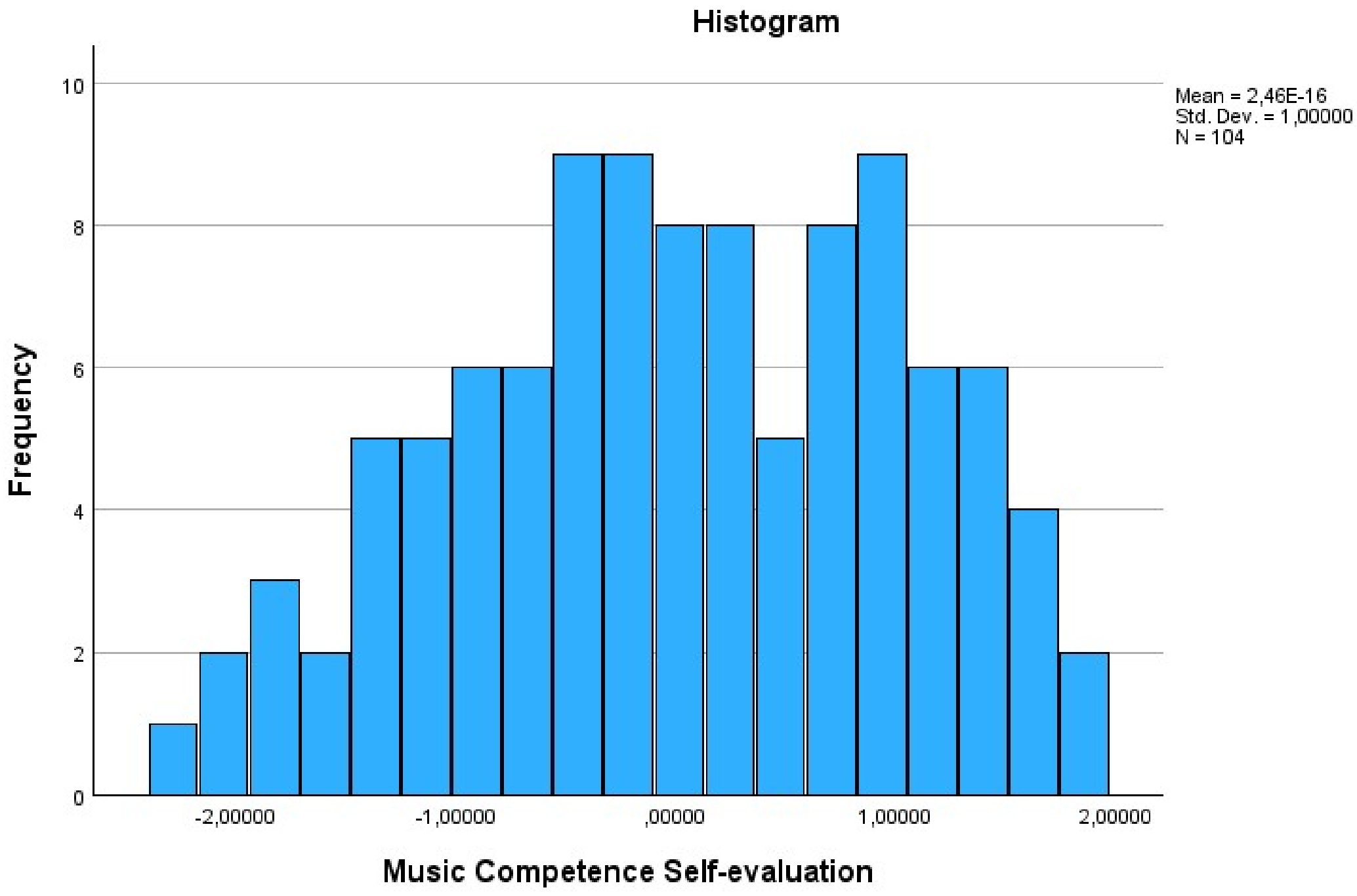

Figure S5. Distribution of the Language Summary Index in the sample.

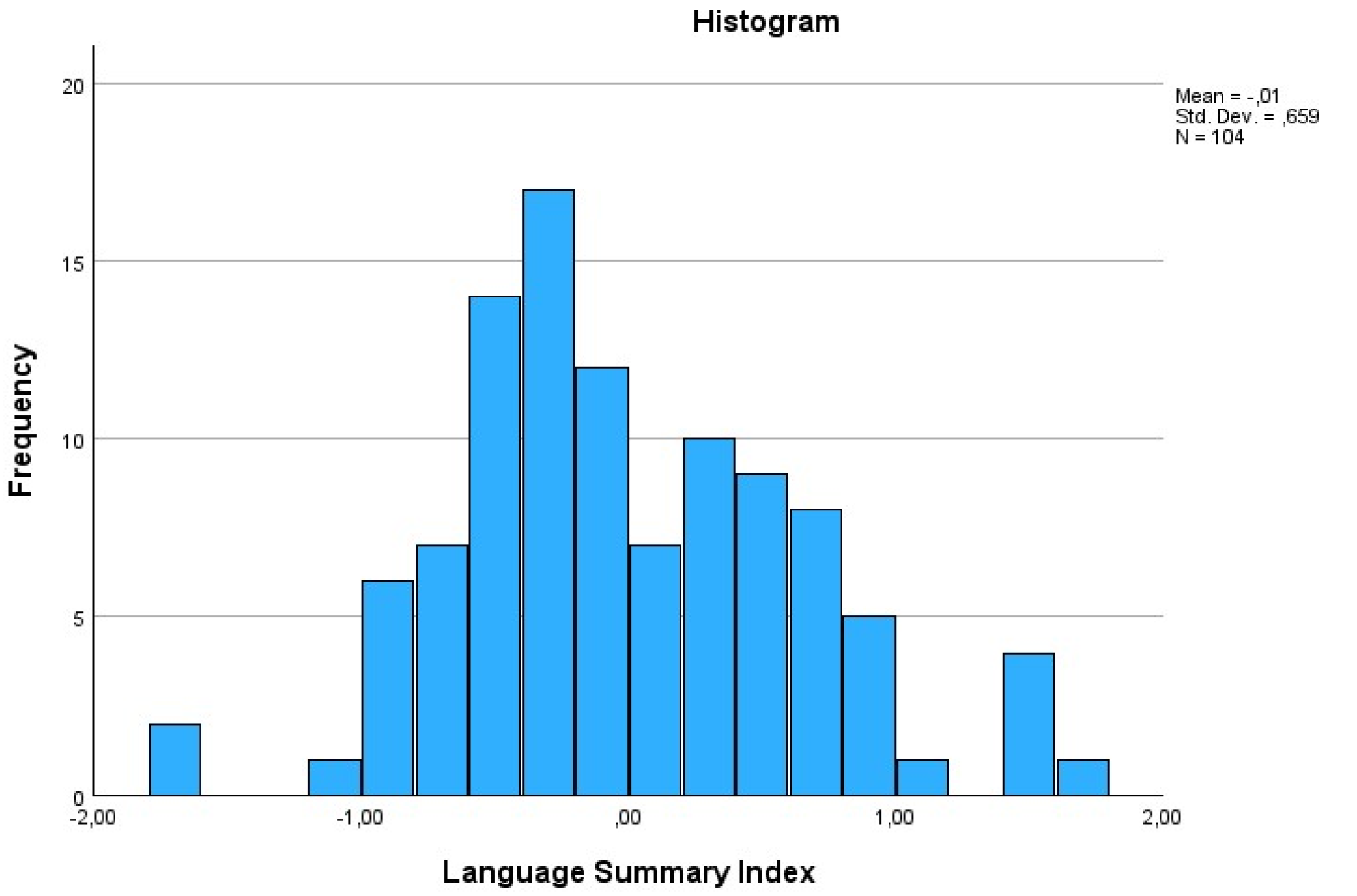

Figure S6. Distribution of the Language Competence Self-evaluation in the sample.

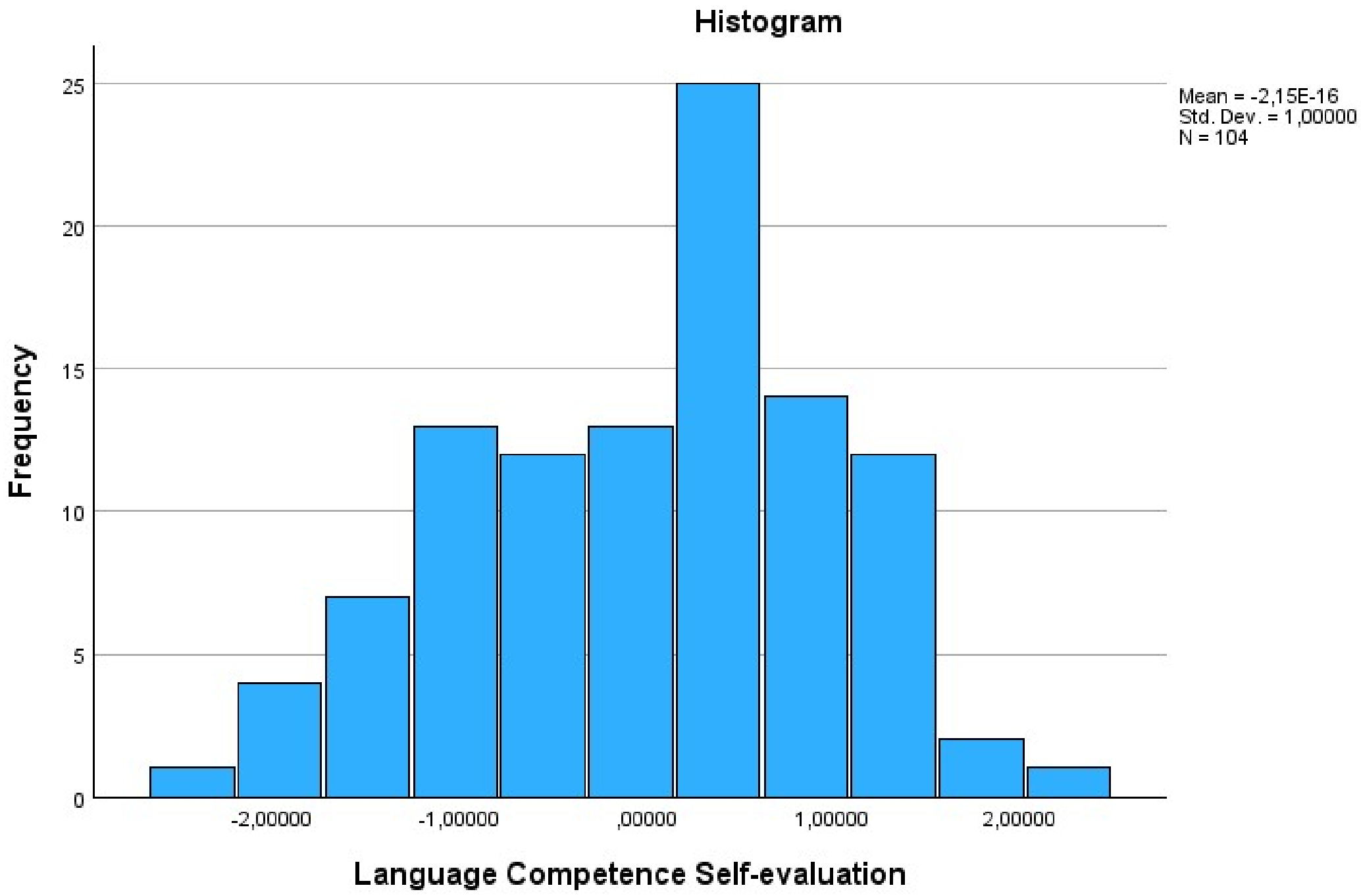

Supplement: Supplementary file 1 [file Data_Sheet_1.pdf]
